# Decoding thymic development: a single-cell atlas of tree shrew immunity

**DOI:** 10.3389/fimmu.2025.1689906

**Published:** 2026-01-16

**Authors:** Haibo Tang, Yunlin He, Lifeng Zhang, Yingying Cao, Baoying Li, Liang Liang, Chengxia Yun, Junyu Tao, Shanshan Zhai, Zhuxin Li, Yinghan Dai, Yanling Hu, Jing Leng

**Affiliations:** 1 Guangxi University of Chinese Medicine, Nanning, China; 2 Guangxi Key Laboratory of Translational Medicine for Treating High-Incidence Infectious Diseases with Integrative Medicine, Nanning, China; 3 Guangxi Henbio Biotechnology Co., Ltd., Nanning, Guangxi, China; 4 Guangxi Health Commission Guangxi Key Laboratory of Molecular Biology of Preventive Medicine of Traditional Chinese Medicine, Nanning, China; 5 Key Laboratory of Characteristic Experimental Animal Models of Guangxi, Nanning, China; 6 Life Sciences Institute, Guangxi Medical University, Nanning, Guangxi, China; 7 Guangxi Zhuang Autonomous Region Engineering Research Center of Graphene Biomedical Application Technology, Nanning, China

**Keywords:** postnatal development, senescence, single-cell, thymic, tupaia belangeri yaoshanensis (T.b.yaoshanensis)

## Abstract

The tree shrew is a potential mammalian model for preclinical studies, but its immune system is not well understood. In this study, we utilized single-cell RNA sequencing (scRNA-seq) to construct a comprehensive cell atlas of the postnatal thymus development of the tree shrew. Our data revealed that the tree shrew thymocytes exhibit conserved features with species-specific variations in cell states and types when compared to those of humans and pigs. We found that tree shrew thymocyte markers are generally intermediate between humans and pigs, with some resembling each species. Some are different from both humans and pigs, such as the expression of the ZNF683 gene, which is not detected until the later stage of CD8aa cells development. The tree shrew thymic cells were classified into 20 types based on gene expression, confirming previous findings of basic thymic immune cells in tree shrews. The results show that immature immune cells were present in a higher proportion in young tree shrews compared to old ones. As tree shrews mature, there was a significant increase in the proportion of late-differentiated functional cells, such as Treg cells. A pseudo-temporal analysis revealed that thymocytes in tree shrews predominantly originated from a common starting point, with the exception of B and dendritic cells. A pseudotime trajectory analysis identified 17 gene modules, aligning with known T cell development stages and markers. Aging-related transcriptional changes in tree shrew thymus cells initially rise and then fall with age. Older tree shrews show increased expression of aging-related genes and decreased or absent anti-aging genes. These findings elucidate key differentiation events in the maturation of thymopoiesis in tree shrews and offer valuable insights into the development of T cells in this species.

## Introduction

Tree shrews (*Tupaia belangeri*) have emerged as a valuable translational model in scientific research, bridging the gap between rodent and non-human primate models and exhibit a closer genetic affinity to primates than to rodents. The thymus is crucial for T-cell development, requiring precise organization to regulate T lymphocyte maturation and selection through various signaling pathways. Tree shrews exhibit greater similarity to humans in terms of physiological functions, biochemical metabolism, and genomic structure compared to rodents like rats, mice, and marmots. The structure of corneal endothelial cells, adrenal cells, pancreatic cells, and other cells in tree shrews are very similar to those of the corresponding human cells (1, 2). The whole genome sequencing of the Chinese tree shrew has been completed, revealing its close genetic relationship to primates through comparative genomic analysis with other species (3). Resembling squirrels in size, tree shrews have a short lifespan of 6–7 years and a brief reproductive cycle, making them manageable and cost-effective for research purposes (4).

Tree shrews in China are predominantly distributed in Southwest China, including Yunnan, Guizhou, Sichuan, Guangxi, and Hainan provinces. Wang (5) delineated these tree shrews into six subspecies based on geographical distribution, morphological characteristics, and morphometric data: *Tupaia belangeri chinensis*, *Tupaia belangeri gaoligongensis*, *Tupaia belangeri modesta*, *Tupaia belangeri tonquinia*, *Tupaia belangeri yunalis*, and *Tupaia belangeri yaoshanensis* (5). The subspecies *Tupaia belangeri yaoshanensis* (*T.b. yaoshanensis*) is distinctively located in the Dayao Mountains of Guangxi, China (106° 38′ E, 24°63′N), situated in the central region of the province (6). The application of tree shrew models in various disease research has demonstrated extensive potential. Their unique genomic characteristics and biological attributes position them as a vital experimental platform in fields such as infectious diseases, metabolic disorders, oncology, and neuroscience. Tree shrews have been confirmed to be susceptible to a variety of human-related viruses, including Epstein-Barr virus (EBV) (7), hepatitis B virus (HBV) (8), hepatitis C virus (9), influenza virus (10), etc. Existing studies have shown that tree shrews not only have advantages in simulating the pathological mechanisms of human diseases, but also the histological characteristics of their immune and endocrine systems closely resemble those of primates (11, 12). Despite the numerous advantages of utilizing tree shrews as a small animal model in immunological research, their immune system characteristics have not been systematically and comprehensively analyzed, particularly concerning cellular immune responses. The types, proportions, and marker molecules of immune cells in tree shrews remain inadequately characterized, and a systematic theoretical framework delineating the specific roles and mechanisms of various immune cell types in tree shrew disease models has yet to be established. There is a notable deficiency in the comprehensive assessment of immune responses and a lack of in-depth research regarding the influence of multiple factors. In particular, the absence of identified marker molecules for different immune cells poses a significant bottleneck to their examination, detailed study, and application.

The thymus plays a crucial role in the initial establishment of the peripheral T cell pool in both animals and humans (13, 14). It serves as an indispensable organ for the homeostatic maintenance of the peripheral immune system. As a primary lymphoid organ, the thymus is where T cells undergo development and extensive education before being exported to the periphery, thereby establishing a functional and effective immune system (15). This organ undergoes degeneration early in life, and the consequent reduction in T cell output has been associated with an increased age-related incidence of cancer, infection, and autoimmunity (16, 17). Thymic involution commences during childhood and reaches its peak around puberty in humans (18). In contrast, thymic involution in mice appears to be more gradual, with evidence suggesting that the thymus can remain functional in aged mice (19–21). Age-related thymic involution is characterized by a disruption of tissue architecture, a reduction in thymic mass, and a consequent decline in thymocyte numbers. Despite its critical importance, our understanding of the molecular framework and transcriptional dynamics underlying thymus development remains limited.

A comprehensive understanding of thymopoiesis is crucial for elucidating the mechanisms underlying cellular immunity and T cell-associated pathologies. Although much of the current knowledge regarding thymus function and cellular composition has been derived from rodent models, it is important to recognize that the dynamics of thymopoiesis and the resultant diversity of mature T cell subsets are species-specific (22). This underscores the necessity of studying the thymus within each individual species. To enhance our understanding of these processes, we have developed a single-cell transcriptomic atlas spanning the lifespan of tree shrews, incorporating data from four thymus samples. In this study, we captured cells from the entire organ and employed single-cell RNA sequencing (scRNA-seq) to develop a comprehensive thymus cell atlas for *T.b. yaoshanensis*. We conducted an analysis of thymus from various age groups, specifically at 3 days, 57 days, 1 year, and 5 years and 3 months. We present the inaugural single-cell sequencing analysis of thymus cells in the tree shrews, offering a foundational scientific basis for future research into the developmental and mechanisms of T cell maturation.

## Materials and methods

### Animals

The *Tupaia belangeri yaoshanensis* specimens described in this study were obtained from the Experimental Center of Artificial Domestication and Breeding at Guangxi University of Chinese Medicine. The animals were maintained in our laboratory following the standard protocol [SYXK(Gui) 2024-0004]. All animal experiments were conducted in accordance with the National Guidelines for the Humane Treatment of Laboratory Animals and were approved by the Animal Welfare and Animal Experimental Ethics Committee of Guangxi University of Chinese Medicine (Approval No. DW20201206-123).

### Tissue processing

Four thymic tissues were surgically obtained from healthy tree shrews ranging in age from 3 days to 5 years (specifically, 3 days, 57 days, 1 year, and 5 years and 3 months). The animals were induced under anesthesia with 3% to 4% isoflurane, they were then maintained under deep anesthesia with 2% to 2.5% isoflurane and died by exsanguination under deep anesthesia. Post-isolation, all tissues were processed immediately following standardized protocols. Thymocytes were aseptically isolated by fragmenting the thymic lobes into small pieces and subsequently passing them through a metal mesh. The resulting cell suspension was filtered through a 70 µm sieve, and cells were collected by centrifugation at 800 g for 3 minutes at 4 °C. The cells were then treated with a 1X red blood cell lysis buffer (Miltenyi Biotec) for 10 minutes at room temperature and subsequently washed once with flow buffer (DMEM containing 2% (v/v) and 2 mM heparin sodium) before proceeding to cell counting.

### H&E staining

The fixed four thymus tissues were processed using conventional paraffin embedding techniques, with section thickness controlled at 4-6 μm. Subsequently, the paraffin sections were stained using the hematoxylin-eosin (HE) method, covered with a cover glass, and sealed with gum.

### Single-cell RNA library preparation

From four samples, a range of 5,802 to 17,491 cells was obtained, with variations attributable to the specific animal source of the tissue or sample. The single-cell RNA sequencing (scRNA-seq) dataset for each sample was generated using a Chromium Controller (10× Genomics, PN1000073, Pleasanton, CA, USA) in accordance with the manufacturer’s protocol. Cell viability was assessed using acridine orange/propidium iodide (AO/PI) staining with a LUNA-FL™ automated cell counter (Logos Biosystems, South Korea), revealing a viability ratio exceeding 85.6% for cells in single-cell suspension. The concentration of the single-cell suspension was adjusted to 700–1200 cells/μL. The suspended cells were then loaded onto a Chromium Controller (10×Genomics, Pleasanton, CA, USA) for the generation of Gel Beads in Emulsion (GEMs). Subsequent cell lysis facilitated the release of cytoplasmic RNA, and all barcoding steps, including reverse transcription, were completed within individual GEMs. Library sequencing was conducted using the Illumina NovaSeq 6000 platform (Illumina, San Diego, CA, USA) with 150-base pair paired-end reads.

### Single-cell RNA sequencing data processing

The processing of single-cell RNA sequencing data involved handling raw files, including previously published datasets of the tree shrews, using Cell Ranger (version 3.0.0, 10×Genomics, Pleasanton, CA, USA) with default mapping parameters. The reads were aligned to the Tree Shrew (Tupaia belangeri) reference genome, accessible at https://asia.ensembl.org/Tupaia_belangeri/Info/Index and http://www.treeshrewdb.org/. Single-cell RNA sequencing (scRNA-seq) data analysis and marker identification were performed using the Seurat toolkit (version 4.1.1) within the R statistical environment (version 4.2.0). Initially, LincRNAs were excluded from the cells. To ensure the inclusion of high-quality cellular data, stringent filtering criteria were applied. Specifically, only cells expressing between 200 and 5000 genes were retained, with mitochondrial gene expression constituting less than 5% of the total gene expression and red blood cell gene expression constituting less than 1% of the total. Ultimately, a total of 36,811 filtered thymocyte cells from four thymus tissues were selected for further analysis. The subsequent analysis included the normalization of the scRNA-seq data for clustering and annotation purposes.

### Construction of reference genomes and annotation files

The sequence alignment of tree shrews necessitates the development of reference genomes and annotation files, specifically the FASTA version of the reference genome and the GTF annotation file. These resources can be accessed at https://asia.ensembl.org/Tupaia_belangeri/Info/Index and http://www.treeshrewdb.org/. The reference genome index file was generated using the “cellranger mkref” command from the Cellranger software (version 7.2.0). Subsequently, the paired FASTQ files (R1 and R2) for each sample were aligned and annotated using the “cellranger count” command. This process resulted in the creation of both the original gene expression matrix file and the filtered gene expression matrix. The filtered gene expression matrix, representing a refined dataset, will be utilized for subsequent downstream analyses.

### Clustering and annotation of scRNA-seq data

To mitigate the influence of technical variations, such as sequencing depth, on the observed biological heterogeneity in single-cell RNA sequencing (scRNA-seq) data, the “NormalizeData,” “FindVariableFeatures,” and “ScaleData” functions were applied sequentially for data normalization and scaling. This process facilitated the identification of highly variable genes for subsequent dimensionality reduction using the “RunPCA” function, which extracts the principal features of the dataset for further analysis and visualization. Subsequently, dimensionality reduction and clustering analysis were conducted on the integrated dataset. The “FindNeighbors” function was employed for cell clustering, followed by the “FindClusters” function to cluster the entire cell population, with a resolution parameter set at 0.8. Subsequently, employ the “RunHarmony” function from the Harmony package (version 0.1.0) to mitigate batch effects across all samples. Implement the ‘RunUMAP’ function to facilitate dimensionality reduction. The “FindAllMarkers” function was utilized to apply the Wilcoxon rank sum test for identifying marker genes specific to each cluster. Genes exhibiting an average log fold change (avg1 logFC) exceeding 0.25 and a p-value less than 0.05 were deemed significantly different. Cell types were determined based on the expression profiles of these characteristic marker genes. Additionally, the Seurat R package (version 5.1.0) facilitated downstream principal component analysis (PCA) and t-distributed stochastic neighbor embedding (t-SNE) analysis. Cluster cell identities were determined through manual annotation using known marker genes and differentially expressed genes (DEGs) identified via a custom R software function. Clusters exhibiting distinct and uniform identities were annotated initially, and a logistic regression model was subsequently trained based on this annotation.

### Alignment of data across different batches

Batch alignment can be influenced by variations in the chemistries applied to the same cell populations, such as the 10×Genomics 5’ and 3’ chemistries, or by differences in donor cells analyzed using the same chemistry. These variations introduce technical or biological discrepancies between batches. To address this, we implemented an iterative batch correction process. Initially, we performed a preliminary alignment of batches across similar samples using the `RunHarmony` function. This batch-aligned manifold facilitated the annotation of cell types. Following a preliminary cell type annotation, we employed an L2-regularized linear model, incorporating batch variables (e.g., 10×chemistry, donor identity) or cell type annotations as categorical variables. We then regressed out the variations attributable to batch effects, retaining the residuals that encapsulate biological information. Subsequently, we realigned the batches using the `RunHarmony` function to achieve a high-resolution, batch-integrated manifold, which was utilized for refined annotation, visualization, and trajectory analysis.

### Estimating cellular composition per sample

If all cell types used for a comparison come from the same sorting gate, we simply calculated the proportion as: number of cells in specific cell type/total number of cells in comparison set. These normalised numbers are used to calculate proportions, which eliminates bias caused by sorting different numbers of cells into different gates. The significance of changes in cellular proportions are tested by t-test on cell proportions. To estimate the relative proportions of various cell types across different samples, we categorized the cells into broad types (e.g., B naive, Treg1 cells, total cells) and calculated the proportion of each cell type within a designated group of cells. When all cell types used for comparison originate from the same sorting gate, the proportion was determined by dividing the number of cells of a specific type by the total number of cells in the comparison set. These normalized values are used to calculate proportions, thereby eliminating biases introduced by sorting varying numbers of cells into different gates. The significance of changes in cellular proportions was assessed using a t-test on the cell proportions.

### Trajectory analysis

To model differentiation trajectories, we employed a combination of linear regression and batch-alignment algorithms, as previously described, to construct a neighborhood graph. The robustness and accuracy of the batch-alignment process were evaluated by comparing multiple batch-alignment methods. Subsequently, diffusion pseudotime was calculated using the learn_graph function in monocle3, initiating from a manually selected progenitor cell. The progenitor cell was chosen from the extremities of the diffusion components. Cells from four thymus tissues are categorized according to pseudotime ordering, and differentially expressed genes are identified as those exhibiting significantly different expression levels compared to a randomly permuted background within any of the bins.

### Comparison to published dataset

The human thymic dataset was obtained from the Gene Expression Omnibus (GSE139042), and the porcine thymus dataset was sourced from GSE192520, both in the form of processed count matrices. These datasets were processed using the same analytical pipeline and subsequently integrated with a tree shrews thymus dataset. Batch alignment across the thymus datasets was conducted using the BBKNN algorithm, supplemented by linear regression.

### Gene orthologue mapping

To map genes between species, we employed the R-based Biological Entity Dictionary (BED) (23). Initially, Ensembl gene names from four tree shrew samples were converted to their human and mouse counterparts. Subsequently, we isolated genes that were common across species, present in the dataset, and identified according to the BED tool’s preferred ID. For genes that did not exhibit a one-to-one correspondence between tree shrew and human or tree shrew and mouse, we retained the gene with the highest expression level in the respective species dataset.

### Age-relevant coefficient of variation analysis

An analysis of age-relevant CV was conducted to examine the effects of aging on various cell types (24, 25). The “Find Variable Genes” function in the Seurat software package was employed to identify highly variable genes (HVGs), with the top 10% of these genes selected for subsequent analysis. Next, the absolute value of the cell-paired-distance dc, x was calculated between each HVGs expression in all cells of the young individuals and the old individuals in each cell type c:


$$\mu_{c,\;x}=|X_{c,\;j}-X_{c,\;j}|;i\in \{1,\;2,\;\&,\;y\},\;j\in \{1,\;2,\cdot \cdot \cdot ,0\}$$

Finally, the arithmetic mean of dc, x (mc, x), and the standard deviation of dc, x as (sc, x) were calculated. Accordingly, the aging-related transcriptional variation of each cell type is defined by the following formula: CVc, x = σ_c, x_/µ_c, x_ × 100.

### Analysis of senescence-related genes in thymocytes of four tree shrews

We compiled a list of aging-related genes from humans (718 genes), mice (489 genes), and model organisms (2205 genes) by referencing three established aging-related resources from prior studies (26–29). After eliminating duplicate or synonymous genes, a comprehensive library of 2729 unique aging-related genes was established. The “FindMarkers” function in Seurat was then utilized to identify genes associated with aging across various age groups (3 days, 57 days, 1 year, and 5 years and 3 months) within each cell type. Only those genes with |”avg_logFC”| > 0.25 and “p_val_adj” < 0.05 are considered age-related differentially expressed genes (DEGs).

## Results

### Cellular composition of the thymus in the tree shrew

To comprehensively characterize the cellular composition of the tree shrew’s thymus at the single-cell level, we constructed single-cell RNA sequencing (scRNA-seq) libraries from four individuals aged at 3 days, 57 days, 1 year, and 5 years and 3 months. The libraries were subjected to high-throughput RNA sequencing. In total, we obtained a scRNA-seq dataset comprised of 36,811 cells, with 11,276 cells from the 3-day-old, 10,069 cells from the 57-day-old, 9,664 cells from the 1-year-old, and 5,802 cells from the 5-year and 3-month-old tree shrews. After excluding cells with anomalously low or high gene counts and elevated mitochondrial gene expression, 36,183 cells including 11,071, 9,940, 9,449, and 5,723 cells at different age groups were retained for further analyses. The data demonstrating the quality control are presented in **Supplementary Figure S1**. Subsequently, unsupervised clustering was performed for the qualified cells and the results are presented in various clusters, visualized using a UMAP plot with a resolution range of 0.1 to 1.5 (**Supplementary Figure S2**). Based on the analysis of cellular subpopulations, cell markers, and the clustering characteristics of thymic cells, a resolution coefficient of 0.8 was ultimately selected for the clustering of thymic cells in the tree shrews.

**Figure 1:**
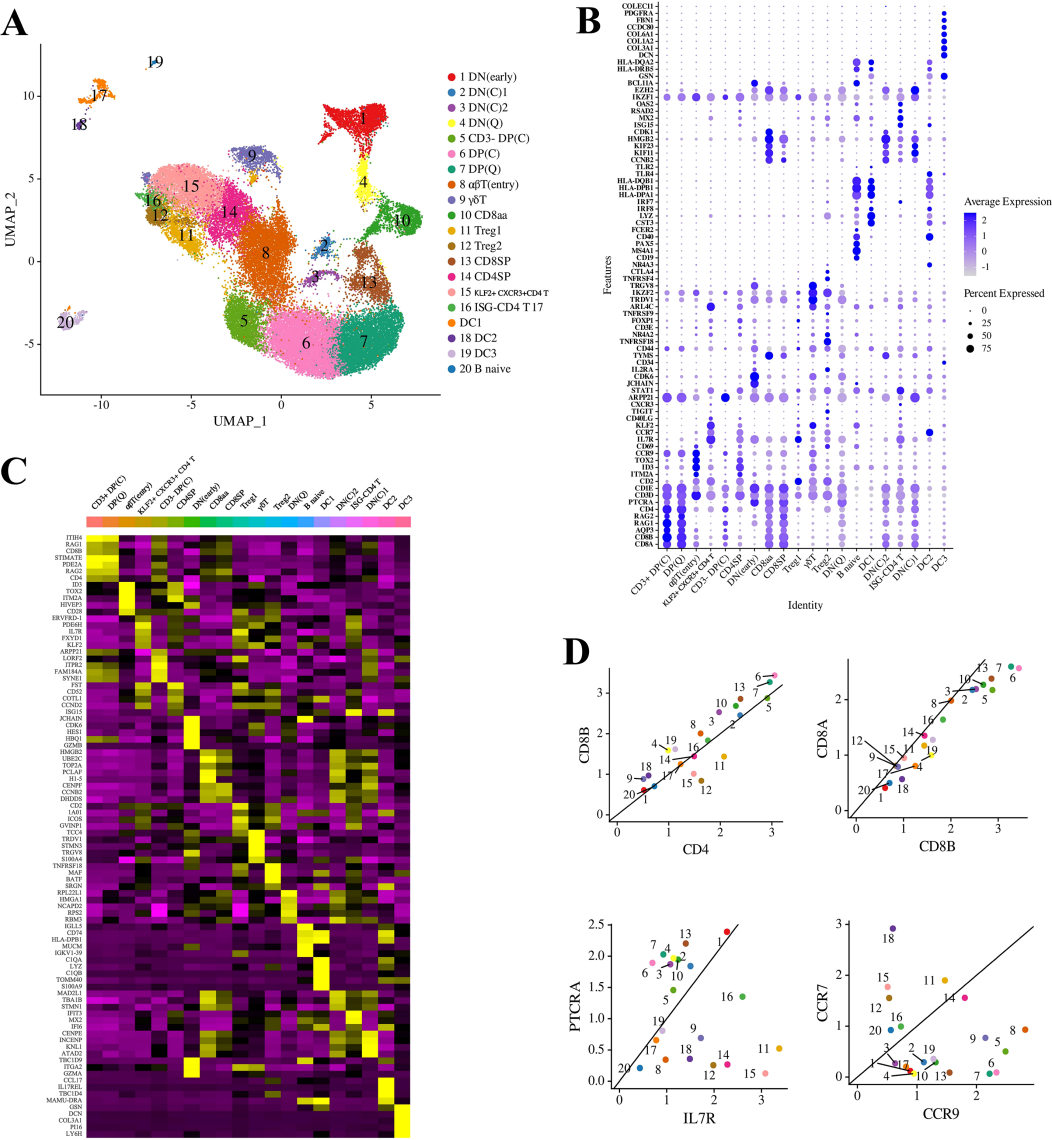
Diversity of cell types identified through single-cell RNA sequencing (scRNA-seq) analysis. **(A)** Uniform Manifold Approximation and Projection (UMAP) visualization of thymic cell types in tree shrews, with clusters color-coded. Clusters were identified using the graph-based Louvain algorithm at a resolution of 0.8. **(B)** Dot plot illustrating the Z-scored mean expression of marker genes utilized for the classification of cell types within the clusters. The intensity of the color reflects the average expression level of each marker gene across the respective clusters, while the size of the dots indicates the proportion of cells expressing each marker gene. Genes exhibiting cluster-specific increases in expression are detailed in **Supplementary Table S2**. **(C)** Heatmap displaying row-scaled mean expression levels of the five most significantly differentially expressed transcription factors within each cluster. **(D)** Scatterplots depicting the ratios of various lineage marker genes across the different thymocyte clusters.

The results showed that thymic cells of the tree shrews could be categorized into 20 distinct cell clusters (clusters 1-20) (**Figure 1A**). Annotation of these cells was conducted based on the expression profiles of cell-type-specific marker genes (**Figures 1B-D** and **Supplementary Figure S3**; **Supplementary Tables S1-2**). The dataset prominently features differentiating T cells, including CD4− CD8− double negative (DN), CD4+ CD8+ double positive (DP), CD4+ single positive (CD4SP), interferon-stimulated gene (ISG)-CD4 T, KLF2+ CXCR3+ CD4 T, FOXP3+ TIGIT+ regulatory T (Treg), CD8+ single positive (CD8SP), CD8αα, αβT(entry), and γδT cells. Previous studies have classified the early T cell population, marked by a strong cell cycle signature, as cycling (C), and the later population as quiescent (Q) (22, 30). Double-negative (DN) and double-positive (DP) cells were further delineated into two phases based on the expression of cell cycle-related genes. In this study, tree shrews DN cells were categorized into bone marrow-derived clusters DN(early) cluster 1, DN(C) (clusters 2–3), and DN(Q) (cluster 4) according to the expression of hematopoietic stem cells and multipotent progenitor cells (HSC/MPP) marker genes, as well as cell cycle and VDJ recombination genes (**Figures 1B** and **Supplementary Figure S3A**) (30). Additionally, other immune cell types such as B cells and dendritic cells (DCs) were identified. B cells were further subclassified into B naive, while DCs were categorized into myeloid/conventional DC1, DC2, and DC3. Clusters 17–19 were annotated as dendritic cells (DC) and characterized by the enriched expression of CST3, LYZ, IRF8, IRF7, HLA-DPA1, HLA-DPA2, HLA-DPB1, HLA-DQB1, and TLR4. Cluster 20 was identified as naive B cells, distinguished by the specific expression of CD19, MS4A1, PAX5, CD40, and FCER2 (**Figure 1A**; **Supplementary Tables S1-2**). Due to the insufficiency of the method for collecting tree shrew thymus cell samples, no thymic epithelial cells or macrophages were found in this study.

### Pseudo-temporal analysis of thymocyte development

**Figure 2:**
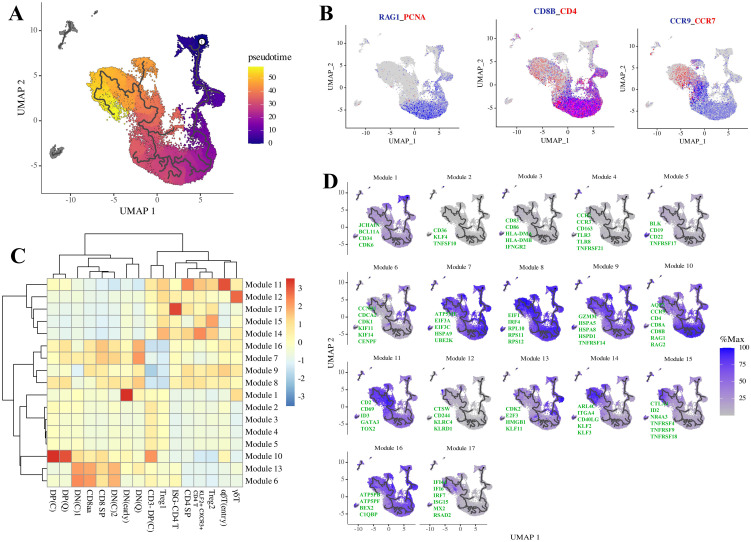
Pseudotemporal analysis of thymocyte development in tree shrews. **(A)** Pseudotime trajectory generated using Monocle 3, incorporating clusters 0–16 as depicted in **Figure 1A**. **(B)** UMAP visualization highlighting classical stage-specific markers associated with thymocyte development. **(C)** Heatmap illustrating the expression patterns of 17 gene modules, which exhibit variation across pseudotime among the clusters. **(D)** UMAP visualizations depicting the expression profiles of selected genes from modules 1–17. Refer to **Supplementary Table S3** for a comprehensive list of genes within each module.

To elucidate the differentiation trajectory of tree shrews thymocytes in greater detail, we performed a pseudotime analysis. This analysis revealed an ordering of cells that aligns closely with known marker genes and stage-specific transcription factors (**Figure 2**). The trajectory commenced with CD4-CD8- double-negative (DN) early-stage cells, which progressively express CD4 and CD8 to become CD3- CD4+CD8+ double-positive (DP) cells. These cells further develop into CD3+CD4+CD8+ DP cells and subsequently transition through a CCR9 high αβT entry stage, ultimately diverging into mature CD4+ or CD8+ single-positive (SP) cells. The final transformation results in Treg and KLF2+ CXCR3+ CD4 T cells. Genes exhibiting variation with pseudotime were organized into 17 distinct modules (**Figures 2C, D**; **Supplementary Table S3**). The pseudotime series plot is color-coded from purple to yellow, indicating the progression from early to late differentiation stages. Module 1 demonstrated high expression of genes associated with bone marrow-derived stem cells, such as CD34, a well-established marker for identifying hematopoietic stem and progenitor cells; CDK6, which is implicated in the proliferation and differentiation of stem cells; and JCHAIN and BCL11A, which intersect with the DN (early) and DN (Q) clusters. Modules 2–5 exhibited subtle variations over pseudotime and did not converge with genes specific to particular cell clusters. Modules 6 and 13 intersected with DN cells (DN(C)1 and DN(C)2), including KIF11, which primarily functions in the formation and maintenance of bipolar spindles during mitosis, as well as cell cycle-related genes such as CCNB1, CDK1, CDK2, and E2F3. Modules 7–9 and 16 displayed high expression levels of ATP synthase genes and core translation initiation genes, which were prominently expressed in DN(Q) clusters. Module 10 intersected with DP cells (CD3- DP(C), DP(C), DP(Q)), showing strong upregulation of genes associated with VDJ recombination, including RAG1 and RAG2, as well as classical DP markers such as CD4, CD8, and AQP3. Module 11 contained marker genes CD2, CD69, ID3, GATA3, and TOX2, which intersected with various post-committed populations, including αβT(entry) and CD4+ T cells. Module 12 intersected with γδT cells and included genes such as CTSW, CD244, KLRC4, and KLRD1. Module 14 intersected with KLF2+ CXCR3+ CD4 T cells and a small subset of Treg1 cells, containing genes encoding memory markers such as ARL4C, ITGA4, CD40LG, KLF2, and KLF3. Module 15 intersects with Treg2 cells and exhibits high expression of signature genes, including CTLA4, TNFRSF4, TNFRSF9, TNFRSF18, ID2, and the Nr4a receptor NR4A3, which are essential for Treg cell lineage commitment in mice (31, 32). Module 17 encompasses several interferon-stimulated genes (ISGs) and demonstrates elevated gene expression levels in ISG-CD4 T cells (**Figure 2D**).

### Characterization of unconventional T cell populations

**Figure 3:**
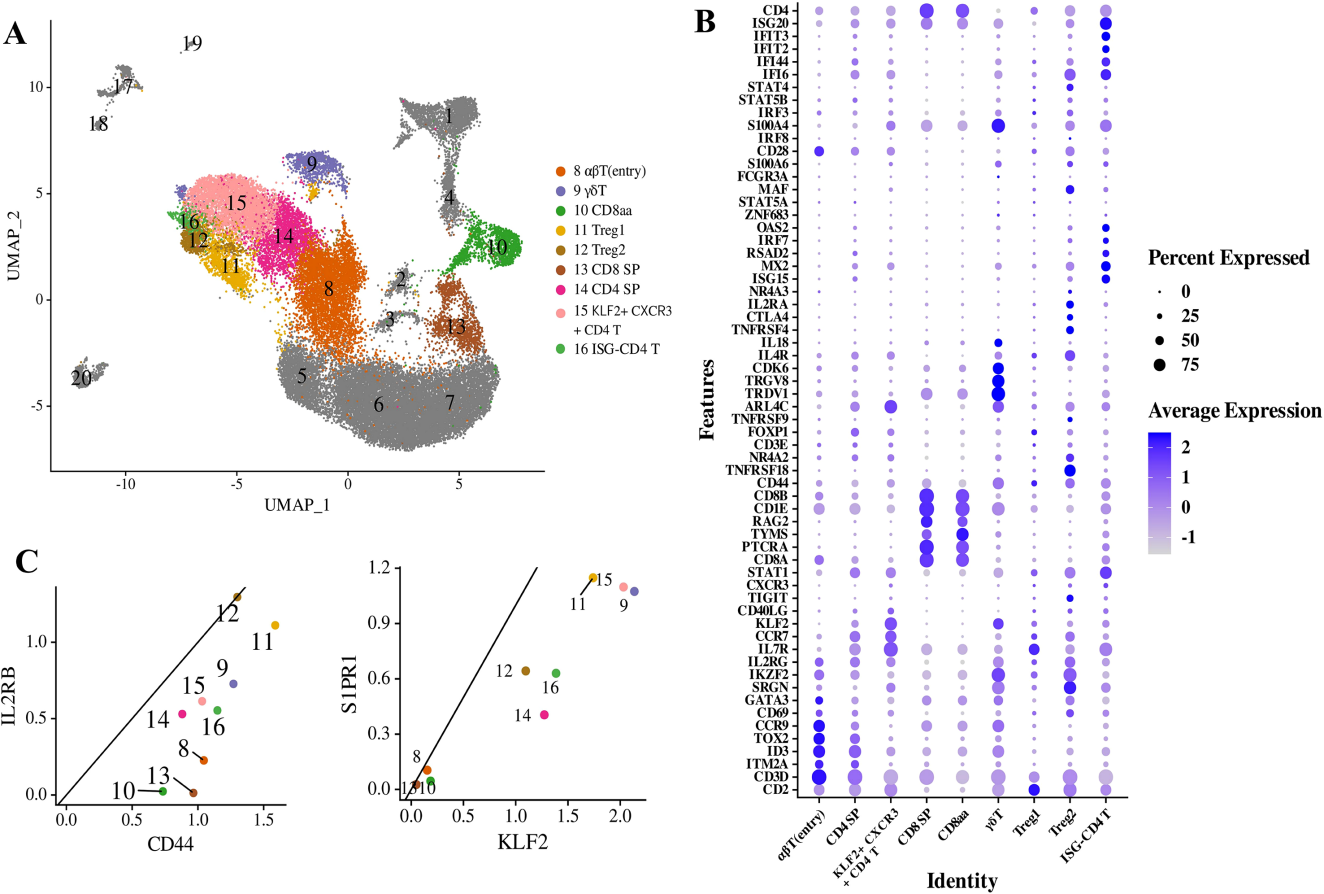
Analysis of unconventional T cell populations in tree shrews. **(A)** UMAP representation of post-committed thymocyte populations. **(B)** Dot plot illustrating Z-scored mean expression levels of selected marker genes within the clusters identified in panel **(A)**. **(C)** Scatterplots depicting the characteristics of mature T cells, comparing gene expression ratios associated with memory T cells (left panel) and thymic emigration (right panel).

In addition to the conventional CD4+ and CD8+ T cells, which represent the predominant T cell populations in the developing thymus, our data have identified several unconventional T cell types characterized by the expression of specific marker genes (**Figure 3**). It is posited that unconventional T cells rely on the thymus and require agonist selection for their development (14). These cells can accumulate in the thymus post-maturation and exhibit a higher relative abundance in the thymus compared to other hematopoietic organs (15). All double-negative (DN) populations exhibited classical DN markers, such as pre-TCRα (PTCRA) (33) (**Figures 1B**; **Supplementary Table S1-2**). The DN(early) subset expressed traditional DN markers, including IL7R, IL2RA(CD25) and Kit (CD117) (34) (**Figures 1B, D**), and showed high expression of hematopoietic stem cell/multipotent progenitor (HSC/MPP) markers, such as CD34 and CDK6 (**Supplementary Table S1**). IL2RA(CD25) serves as a distinguishing factor for DN subpopulations in mice and is upregulated during the early development of tree shrews thymocytes DN(early), but its expression was not detected in either DN (C) or DN (Q) cells. Two rapidly cycling cell clusters, DN(C)1 and DN(C)2 (clusters 2–3), exhibited upregulation of G2M and a combination of G2M- and S-phase cell cycle genes, respectively (**Supplementary Figure S3B**). The DN(C) clusters, characterized by preferential expression of cyclin B2 (CCNB2), cell cycle kinase (CDK1), KIF11, and KIF23 (**Figures 1B**; **Supplementary Table S1-2**), were identified as proliferating DN(C) cells. Double-positive (DP) thymocytes (clusters 5–7) comprised one CD3-negative cluster (CD3- DP(C)), one cycling cluster (CD3+ DP(C)), and one quiescent cluster (CD3+ DP(Q)).

Human thymocytes were found to diverge into two distinct cell lineages at the DN-DP junction, corresponding to the differentiation pathways of αβT (entry) cells and γδT cells (30). Cluster 8 was identified as αβT entry cells due to the high expression levels of CD2, CD3D, CD5, CCR9, ITM2A, ID3, and TOX2, and the low expression of CCR7. Notably, there are significant similarities in gene expression between αβT (entry) cells and γδT cells in tree shrews. Cluster 9 was annotated as γδT cells, characterized by the enriched expression of TRDV1, TRGV3, and TRGV8, which are the most frequently utilized TCRδ and TCRγ V genes in humans, respectively (**Supplementary Table S1-2**). Our observations indicate that γδT thymocytes exhibit an enrichment of genes associated with TCR signaling, such as PRKCH and IKZF2, suggesting a dependence on TCR-mediated stimulation. Furthermore, tree shrews γδT cells express the thymic egress signature genes KLF2 and S1PR1 (34, 35). This evidence suggests that γδT cells are not a thymus-resident population. Cluster 10 was predicted as CD8αα due to the specific high expression of CD8A and low expression of CD8B. However, markers of CD8αα subsets in humans, such as ZNF683 and MME (36), were not detected in tree shrews thymic cells.

Additionally, three unconventional CD4+ T cell subsets were identified, which we designated as Tregs, KLF2+ CXCR3+ CD4 T, and interferon-stimulated gene (ISG)-CD4 T cells. Tregs are among the most abundant unconventional T cells in the thymus of tree shrews. This population expressed Treg markers, including FOXP3, CTLA4, TIGIT, TNFRSF18 and IL2RA (CD25) (**Figures 3**; **Supplementary Table S1, S2, S10**). The development of Treg cells aligns with a previously described two-step model of murine Treg cell development (31, 37). The first step is driven by strong TCR stimulation, generating Treg cell progenitors that express high-affinity CD25 and members of the TNF receptor (TNFR) superfamily, such as GITR, OX40, and TNFR2. The second step relies on cytokine signals that promote Treg cell maturation by phosphorylating Stat5 and upregulating FOXP3. Compared with canonical human mature regulatory T cells (Tregs), the Treg cells of tree shrews highly express the FOXP3 gene. Meanwhile, the expression levels of TNFRSF9, TNFRSF18 (GITR), TNFRSF4 (OX40), IL2Rβ, STAT1, CD40LG, TIGIT and CTLA4 also increased (**Figure 3B**; **Supplementary Tables S1, S2, S10**). These genes have been implicated in autoimmunity and Treg differentiation (38). Additionally, this subset expressed several members of the Nr4a nuclear receptor family, including NR4A2 and NR4A3 (**Supplementary Table S1-2**), which are essential for converting high-affinity TCR signals into FOXP3 expression (32). Tree shrews Treg cells also expressed KLF2 and S1PR1, genes associated with thymic egress, suggesting that Treg cells undergo differentiation in the thymus before migrating to the periphery to fulfill their functional roles.

KLF2+CXCR3+ CD4 T cells expressed a high ratio of CD2, CCR7, IL7R, CD40LG, and CD69, which are conventional CD4SP cell markers (**Figure 3B**; **Supplementary Tables S1-2**). The effector memory CD4+ T cells (CD4+ Tem) were characterized by high expression levels of CCL5, CXCR3, IL7R, CCR7, and SELL (39). Compared with conventional CD4SP cells, KLF2+CXCR3+ CD4 T was enriched for T cell memory markers IL2RB, CXCR3, SELL and ICOS (**Figure 3B**; **Supplementary Table S1, S2**). These tree shrews KLF2+CXCR3+ CD4 T cells highly expressed of KLF2 and S1PR1, which are required for thymic egress (33, 35). This suggests that KLF2+CXCR3+ CD4 T cells undergo differentiation in the thymus and subsequently migrate to the periphery to fulfill their functional roles. Furthermore, a comparative analysis of gene expression between KLF2+CXCR3+ CD4 T cells and CD4+ T cells revealed that the top 10 highly expressed genes ERVFRD-1, PDE6H, IL7R, FXYD1, KLF2, GIMAP7, SARDH, CCND2, SMAD7, and CAV2 were upregulated in KLF2+CXCR3+ CD4 T cells (**Figure 3B**, **Supplementary Table S1-2**).

Previous studies have demonstrated that type I interferon (IFN) signaling plays a role in the induction of interferon-stimulated genes (ISGs) during the later stages of CD4+ and CD8+ thymocyte development in both humans and mice, even in the absence of infection (40). In the present study, ISG-expressing CD4+ T cells in tree shrews were characterized by the expression of markers such as CD2, CCR7, IL7R, CD40LG, and CD69, displaying expression patterns closely resembling those of conventional CD4+ T cells. Notably, we identified the presence of ISG-expressing CD4+ T cells in tree shrews, but did not observe ISG-expressing CD8+ T cells, a finding that contrasts with the formation of ISG-expressing CD8+ T cells in the thymus of pigs (22). The ISG-expressing CD4+ T cells in tree shrews were enriched with ISGs, including MX2, ISG15, RSAD2, OAS2, IRF7, STAT1, interferon alpha-inducible protein 6 (IFI6), IFI44, and interferon-induced proteins with tetratricopeptide repeats 2 (IFIT2) and IFIT3, which are known to mediate the antiviral effects of IFN-alpha and type I IFN signaling (**Figure 3B** and **Supplementary Table S1-2**). Comparative analysis of gene expression between ISG-expressing CD4+ T cells and conventional CD4+ T cells revealed that the upregulated genes in ISG-expressing CD4+ T cells are predominantly associated with anti-infective immune responses. The top ten highly expressed genes in ISG-CD4 T cells were identified as SLFN5, ISG15, IFIT3, IFIT2, RSAD2, EIF2AK2, DDX60, IRF7, MX2, and OAS2 (**Figure 3B**, **Supplementary Table S1-2**).

### Conservation of thymocyte signatures across human, pig, and tree shrews

**Figure 4:**
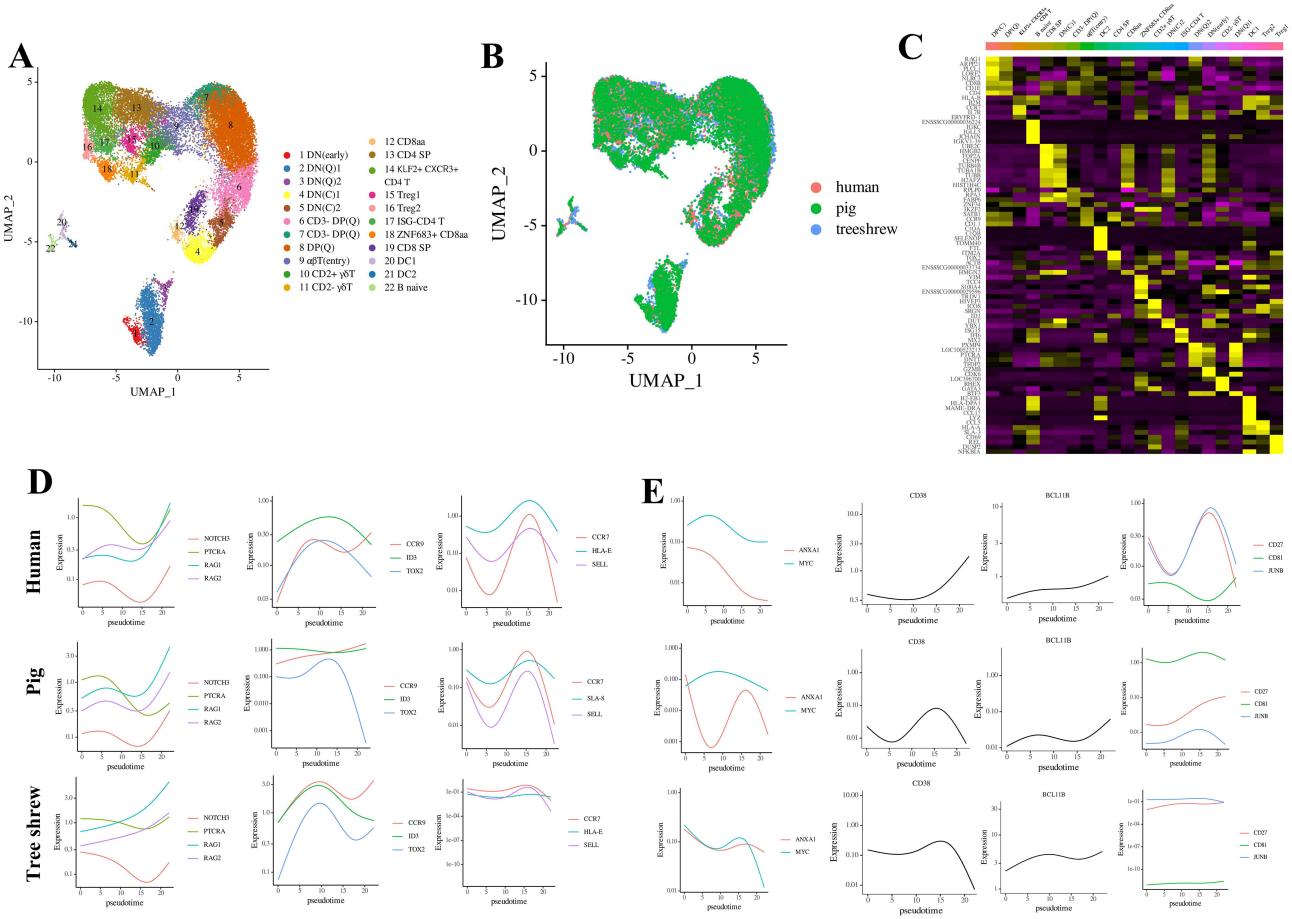
Comprehensive analysis of thymocytes from humans, pigs, and tree shrews. **(A)** UMAP visualization depicting an integrative analysis of thymocytes from humans, pigs, and 57-day-old tree shrews, utilizing canonical correlation analysis to identify shared genes across datasets. **(B, C)** Transcription factors and lineage-specific genes exhibiting conserved **(B)** and divergent **(C)** transcriptional profiles among pigs, humans, and tree shrews.

To compare the transcriptional landscapes of thymocytes in tree shrews, humans, and pigs, we integrated two published single-cell RNA sequencing (scRNA-seq) datasets: one from CD34+ thymic cells of a 19-month-old human (41) and another from combined thymic cells of two 22-week-old mixed-breed pigs (22). These datasets were analyzed alongside our dataset from 57-day-old tree shrews. Our analysis revealed a substantial degree of overlap among most cell clusters (**Figure 4B**). Across all three species, we identified 22 primary cell types (clusters 1-22) (**Figure 4A**), which were annotated based on lineage marker genes that differentiate thymocyte subsets in mice, pigs, and humans (**Figures 4C** and **Supplementary Figure S4A**; **Supplementary Table S4-5**). The newly annotated cell population included several additional cell clusters compared to tree shrews thymic cells alone, such as ZNF683+ CD8aa cells and an additional CD2- γδT cell cluster. In tree shrews, the CD3- DP(Q), DP(C), DP(Q), and CD2+ γδT cell clusters were significantly larger. Conversely, the DN(early), DN(Q)2, and CD2- γδT cell clusters in tree shrews were comparable to those in humans and smaller than their counterparts in pigs (**Supplementary Figure S5**). Notably, the cluster of pig CD8aa cells did not overlap with the corresponding clusters in humans and tree shrews, instead occupying a distinct position. This pig CD8aa cell cluster exhibited a mixed αβT (entry) and γδT cell signature and was situated adjacent to both late double-positive (DP) stage cells and late αβT (entry) cells (**Supplementary Figure S5**).

To examine temporal variations in gene expression across different species, we performed a comparative analysis focusing on key genes involved in thymocyte development (**Figure 4D**). The gene profiles that are conserved, such as those associated with T lineage (NOTCH3 and PTCRA), VDJ recombination (RAG1 and RAG2), DP-to-SP transition (ID3, TOX2, and CCR9), cell cycle regulation (CDK2), and terminal differentiation (CCR7, MHC I, and SELL), which have been previously documented in pigs and humans (22), were also highly consistent in the thymus cells of tree shrews (**Figure 4D**). However, other conserved gene profiles, such as those related to cell cycle (PCNA) and terminal differentiation (CD7), exhibited different temporal change patterns in tree shrews thymocytes compared to pigs and humans. This suggests that gene markers conserved across multiple species may not be universally applicable.

The previously reported divergences, including MYC, a transcription factor essential for thymocyte proliferation and differentiation (42); ANXA1 (Annexin-A1), a phospholipid-binding protein involved in modulating the strength of TCR signaling during thymic selection (43); CD27, which influences T cell survival and memory formation (44); CD81, which modulates TCR signaling (45); and JUNB, a component of the transcription factor AP-1 necessary for T cell differentiation (46), were also differentially expressed at the same cell stage in tree shrews thymocytes (**Figure 4E**), as previously observed in pigs and humans (22). Additionally, CD38, a cyclic ADP ribose hydrolase responsible for inducing apoptosis of DP thymocytes (47), was highly expressed in human thymocytes at the DP stage. In contrast, this gene was undetected in pig thymocytes until the mature T cell stage, where it was expressed at low levels, a pattern that was also consistently observed in tree shrews thymocytes. The transcription factor BCL11B, recognized as a crucial regulator of thymocyte differentiation and survival (48), is maintained at elevated levels until the commitment stage in humans. However, the BCL11B is infrequently detected in porcine thymic cells, and the expression pattern of this gene in tree shrews aligns with that observed in humans.

To facilitate a direct comparison of gene expression across species at the cellular level, we employed a combination of homology-based, *de novo*, and transcriptome-based methodologies to predict protein-coding genes within the TS_2.0 assembly. This approach led to the identification of 21,043 non-redundant protein-coding genes in tree shrews, a finding that is largely consistent with the previously reported 23,568 non-redundant protein-coding genes in this species (49).

Additionally, genes with the suffix ‘li+’ were excluded from this dataset. Further refinement of the data was conducted by applying specific criteria: cells expressing fewer than 200 genes were excluded, and genes were retained only if expressed in at least three cells. This process yielded the number of effectively expressed genes available for subsequent analysis. Among approximately 15,876 human, 13,820 pig, and 13,013 tree shrew genes, 8,670 genes were found to be mappable across all three species. These 8,670 genes accounted for an average of 79.8% of the total variance within each species (human = 73.46%, pig = 79.94%, tree shrew = 86.04%).

**Figure 5:**
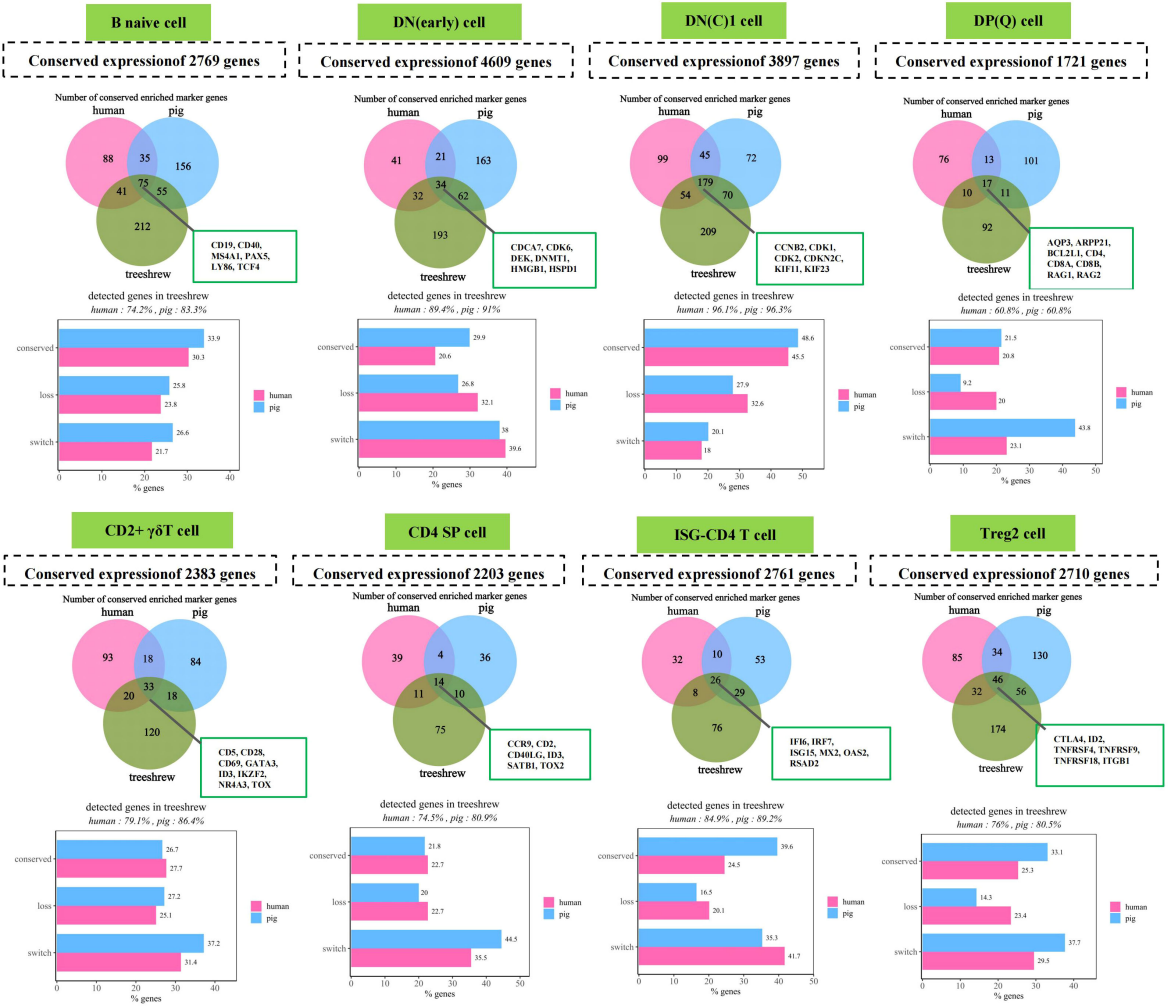
Conservation of marker signatures in thymocytes of humans, pigs, and tree shrews. The upper panel presents a Venn diagram illustrating the overlap of enriched marker genes across species for each cell type. Only marker genes that are mappable across species are included. Selected known overlapping cell type markers and the number of genes exhibiting conserved expression are highlighted. Enriched marker genes are characterized as those expressed in more than 5% of cells within the corresponding cell type and demonstrating increased expression relative to all other cell types (log2-fold change > 0.5). The lower panel depicts the conservation of tree shrew-enriched marker genes in human and pig cell types. The percentage of tree shrew-enriched marker genes that are expressed or detected is indicated. Categories include: “Conserved,” where the enriched marker is present in the same cell type as in tree shrews; “Loss,” where the gene is detected but not as an enriched marker; and “Switch,” where the enriched marker is present in a different cell type compared to tree shrews.

Subsequently, we assessed the overlap in gene expression among species within each cell type (**Figure 5** and **Supplementary Figure S6**). Our analysis revealed that, on average, 2,851 out of 8,670 mappable genes exhibited conserved expression in more than 5% of cells within each thymocyte type across the species studied. This finding suggests that only 30%~40% of the genes expressed in tree shrew cell types are shared with their human and pig counterparts. The majority of the remaining 40%~50% of genes were either expressed exclusively in a different cell type (“loss of expression”) or were not expressed or detected at all. Additionally, 7%∼20% of the genes were not expressed in tree shrews but were detected in human or pig cells (“gain of expression”). These differences may arise from variations in data types, definitions of conservation, or detection limits inherent in single-cell RNA sequencing (scRNA-seq) data. Beyond examining global gene expression profiles, we concentrated on cell type-enriched marker genes to estimate the conservation of cell type-specific functions. In our study, we identified that numerous conserved genes also serve as cell-type marker genes across all examined species. Specifically, these include CD19, CD40, MS4A1, PAX5, LY86, and TCF4 for B naive cells; CDCA7, CDK6, DEK, DNMT1, HMGB1, and HSPD1 for DN(early) cells; CCNB2, CDK1, CDK2, CDKN2C, KIF11, and KIF23 for DN(C)1 cells; AQP3, ARPP21, BCL2L1, CD4, CD8A, CD8B, RAG1, and RAG2 for DP(Q) cells; CD5, CD28, CD69, GATA3, ID3, IKZF2, NR4A3, and TOX for CD2+ γδT cells; CCR9, CD2, CD40LG, ID3, SATB1, and TOX2 for CD4 SP cells; IFI6, IRF7, ISG15, MX2, OAS2, and RSAD2 for ISG-CD4 T cells; and CTLA4, ID2, TNFRSF4, TNFRSF9, TNFRSF18, and ITGB1 for Treg2 cells (**Figure 5** and **Supplementary Figure S6**). Our findings indicate that less than 52.7% of the tree shrew markers were conserved across all cell types, while approximately 9.20% to 41.4% were expressed but did not function as marker genes (‘loss’), and 14% to 49% were markers for different populations (‘switch’). The remaining genes were either undetected or not expressed. These observations align with previous research, which suggests that although essential identity and function marker genes are conserved, cell type-specific expression exhibits greater evolutionary variability.

### The proportion of thymus cells in tree shrews

To enhance our understanding of these processes, we have developed a single-cell transcriptomic atlas spanning the lifespan of tree shrews, incorporating data from four thymus samples. Our analysis revealed cell-specific alterations across multiple cell types, as well as age-related changes in the cellular composition of different thymocyte populations in tree shrews. Age-related thymic involution in tree shrews is characterized by disrupted tissue architecture, reduced thymic mass, and a significant increase in the fat component within thymic tissue in older tree shrews. The thymic lobules become interconnected islands, with indistinct cortical and medullary structures, reduced lymphocyte presence, and lymphocytes appearing scattered or in small clusters. Additionally, the number of thymic corpuscles is significantly diminished (**Supplementary Figure S7**). Therefore, we hypothesize that the number of thymocytes decreases during thymic involution. In this study, we observed that the composition ratio of various cell types in the thymus of tree shrews changes systematically with age. The proportion of naïve T cells, including DN, DP, and CD8+ SP cells, is higher in young animals. As tree shrews age, the proportions of DN(C)1, DN(C)2, DN(Q), and CD8+ SP cells gradually decline (**Supplementary Figure S8**). For instance, the percentage of DN(C)1 cells in infant, juvenile, adult, and elderly tree shrews was 1.86%, 0.52%, 0.31%, and 0.47%, respectively. Similarly, the percentage of DN(C)2 cells was 2.52%, 0.42%, 0.41%, and 0.31%, respectively, and the percentage of CD8+ SP cells was 4.66%, 3.83%, 2.66%, and 1.92%, respectively. The age-related trends of DP(C) and DP(Q) cells parallel those of DN cells, although they peak during adolescence. For example, the percentage of DP(C) cells in infant, juvenile, adult, and elderly tree shrews was 19.95%, 24.17%, 17.50%, and 12.19%, respectively (**Supplementary Table S6**).

The proportion of naïve T cells in young tree shrews was significantly higher than in their older counterparts. With advancing age, there was a notable increase in the proportion of late-differentiated T cells. Significant differences were observed in Treg and KLF2+ CXCR3+ CD4 T cell populations among tree shrews of varying ages, with the proportion of Treg cells markedly increasing with age (**Supplementary Figure S8**). Specifically, the percentages of Treg1 cells in infant, juvenile, adult, and elderly tree shrews were 1.68%, 1.39%, 4.09%, and 7.20%, respectively. Additionally, KLF2+ CXCR3+ CD4 T cells exhibited an upward trend with increasing age. The percentages of KLF2+ CXCR3+ CD4 T cells in infant, juvenile, adult, and elderly tree shrews were 7.27%, 7.29%, 12.27%, and 20.52%, respectively. This suggests that KLF2+ CXCR3+ CD4 T cells in tree shrews are a group of cells closely related to age. Furthermore, the percentages of CD4+ T cells in infant, juvenile, adult, and elderly tree shrews were 4.04%, 5.55%, 7.64%, and 7.58%, respectively. This leads to a reduced production of naive T cells that migrate to the periphery, accompanied by a compensatory clonal expansion of memory T cells and a consequent decrease in the diversity of the peripheral T cell repertoire, thereby impairing pathogen detection. Additionally, alterations in the composition ratios of certain cell types were observed. Notably, the proportion of dendritic cells (DCs) and naive B cells increased with age during the early stages, peaked at maturation, and subsequently declined significantly in aged tree shrews. Specifically, the percentages of naive B cells in infant, juvenile, adult, and elderly tree shrews were 0.86%, 0.90%, 3.00%, and 1.64%, respectively (**Supplementary Table S6**).

### Global transcriptional changes during tree shrews thymus aging

**Figure 6:**
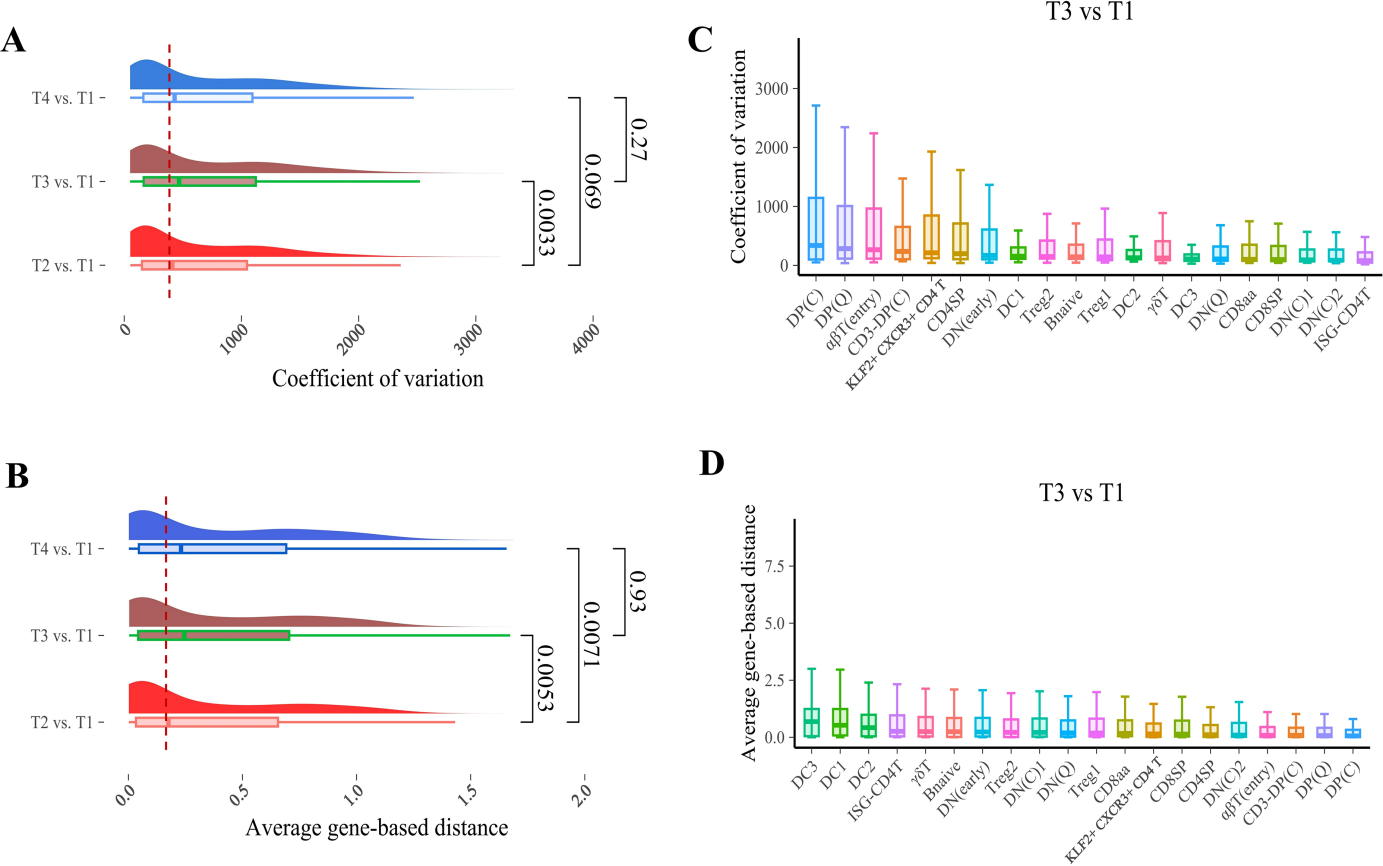
Presents a coefficient of variation (CV) analysis of thymocytes in tree shrews across various cell types. **(A)** A ridge plot illustrates the age-related shift in CV of thymocytes, with p-values determined by the Wilcoxon test. The plot depicts the CV for each cell type across three age comparisons: 5 years and 3 months versus 3 days (top), 1 year versus 3 days (middle), and 57 days versus 3 days (bottom). **(B)** The average of all pairwise distances for each cell type is shown for the same age comparisons: 5 years and 3 months versus 3 days (top), 1 year versus 3 days (middle), and 57 days versus 3 days (bottom). **(C)** A box plot compares the CV of each cell type at 1 year of age to that at 3 days of age, displaying the median and interquartile range (25%–75%), with whiskers extending to 1.5 times the interquartile range. **(D)** The coefficient of variation is calculated as the standard deviation of the distances between genes within cell clusters, divided by the average of these distances, for the ages of 1 year and 3 days.

One of the most thoroughly documented alterations in the immune system associated with aging is the involution of the thymus, the primary organ responsible for generating a highly diverse yet selectively curated T cell repertoire. Thymic involution commences in childhood and reaches its peak around puberty in humans (18). In mice, this process appears to be more gradual, with evidence suggesting that the thymus remains functional in older mice (19–21). However, the age-related involution of the thymus in tree shrews remains unexplored. To elucidate transcriptional perturbations associated with aging in the thymus of tree shrews, we conducted an analysis of the overall coefficient of variation (CV). Our findings indicate that the CV of gene-based distances initially increases and then decreases with age, as evidenced by elevated CV values at 57 days, 1 year, and 5 years and 3 months compared to 3 days old tree shrews, with the CV peaking at 1 year of age (**Figure 6A**). In pairwise age comparisons, the comparison between 1-year-old and 3-day-old tree shrews exhibited the highest CV with a statistically significant difference (p < 0.05). Although CVs increased in other age groups, these differences were not statistically significant. In addition, our analysis revealed that the average gene-based distance in the thymus exhibited a trend similar to that of the coefficient of variation (CV). Specifically, comparisons between 1 year and 3 days, as well as between 5 years and 3 months versus 3 days, demonstrated significantly greater average gene-based distances (p < 0.05) (**Figure 6B**). Upon examining the CV across all thymus cell types between 1 year and 3 days, we identified DP(C), DP(Q), αβT(entry), CD-DP(C), and KLF2+ CXCR3+ CD4 T as the top five cell types with the highest CV values. These elevated CV values suggest that these cell types are more susceptible to aging-related stress compared to others. Conversely, the cell types with the lowest CV values were ISG-CD4, DN(C)2, DN(C)1, CD8 SP, and CD8aa (**Figures 6C, D**). For other age comparisons, such as 5 years and 3 months versus 3 days, and 57 days versus 57 days, the CV of thymus cells exhibited a trend consistent with that observed in the 1 year versus 3 days comparison (**Supplementary Figure S9**).

### Senescence genes expression varies among tree shrew different age groups

To investigate the expression and dynamics of senescence-related genes in thymic cells of tree shrews across different age groups, we have constructed a comprehensive senescence gene library comprising 2,729 genes, based on existing literature. Utilizing this gene library, we conducted a single-cell level analysis to identify senescent genes in the thymocytes of tree shrews. Our findings revealed that a total of 591 senescent genes were expressed in the thymus of four tree shrews (**Supplementary Table S7**). The analysis indicated an age-related increase in the number of expressed senescence genes. Specifically, the number of senescent genes expressed in infant, juvenile, adult, and elderly tree shrews were 465, 443, 489, and 490, respectively. Notably, distinct differences in gene expression profiles regulating aging were observed, particularly in infant and elderly tree shrews, each exhibiting unique patterns of gene expression.

The study delineates the differential expression of genes associated with aging, primarily observed in the distinct genetic profiles of infant and elderly tree shrews. Notably, numerous aging-related genes are expressed in elderly tree shrews that are absent in other age groups. A significant increase in the number of senescent genes was observed in tree shrews aged 1 and 5 years (**Supplementary Table S7**). Subsequently, we conducted an analysis of senescent gene expression across various cell types. Our findings indicate that the expression of senescence-associated genes exhibits distinct patterns at the single-cell level in tree shrews of varying ages. Generally, the expression of most senescence genes increased with age in cell types such as DN(Q), CD3- DP(C), DP(C), DP(Q), αβT(entry), CD8aa, CD8 SP, KLF2+ CXCR3+ CD4 T, and B naive cells. Conversely, in other cell types, the expression of most senescence genes decreased with age, including DN(C)1, DN(C)2, CD4 SP, ISG-CD4 T, Treg1, Treg2, DC1, DC2, and DC3 cells. Furthermore, in certain cell types, the expression of senescence-associated genes, such as DN (early) and γδT cells, increases with age, while the expression of approximately half of these senescence genes decreases with age (**Supplementary Table S8**). In the context of gene expression alterations during aging, much research has concentrated on genes that become upregulated over time. However, there is a comparatively limited understanding of genes that are actively expressed during childhood but become downregulated or even cease to be expressed as individuals age. To address this gap, we utilized the STRING database (http://string-db.org) to predict the interactions and signaling pathways of senescence genes (**Supplementary Table S9**) whose expression diminishes with age. Our analysis revealed significant variability in the reduction of gene expression with age across different immune T cell types, as well as notable differences in the cellular functions these genes are involved in.

Based on the strength of correlation, the principal signaling pathways associated with B cells are as follows: positive regulation of apoptotic signaling pathway (GO:2001235), vascular-associated smooth muscle cell proliferation (GO:1990874), vascular-associated smooth muscle cell migration (GO:1904738), negative regulation of intracellular signal transduction (GO:1902532), and regulation of intracellular signal transduction (GO:1902531). In DN(C)1 cells, the primary signaling pathways include telomere organization (GO:0032200), chromosome organization (GO:0051276), telomere maintenance (GO:0000723), regulation of chromosome organization (GO:0033044), and the cell cycle (GO:0007049). For CD3- DP(C) cells, the key signaling pathways encompass positive regulation of transcription from RNA polymerase II promoter in response to chemical stimuli (GO:1901522), adrenal gland development (GO:0030325), transforming growth factor beta receptor signaling pathway (GO:0007179), endocrine system development (GO:0035270), and SMAD protein complex assembly (GO:0007183). The primary signaling pathways associated with γδT cells encompass DNA topological change (GO:0006265), DNA conformation change (GO:0071103), chromosome organization (GO:0051276), chromatin organization (GO:0006325), and the mitotic cell cycle process (GO:1903047). In contrast, the predominant signaling pathways in CD4 SP cells involve cytoplasmic translation (GO:0002181), structural constituents of ribosomes (GO:0003735), ubiquitin protein ligase binding (GO:0031625), heterocyclic compound binding (GO:1901363), and organic cyclic compound binding (GO:0097159). Furthermore, the key signaling pathways in KLF2+ CXCR3+ CD4 T cells include the cellular response to angiotensin (GO:1904385), regulation of hormone biosynthetic processes (GO:0046885), positive regulation of transcription from RNA polymerase II promoter in response to hypoxia (GO:0061419), positive regulation of cell migration (GO:0030335), and positive regulation of blood vessel endothelial cell migration (GO:0043536). The principal signaling pathways associated with ISG-CD4 cells encompass the mitotic cell cycle checkpoint signaling (GO:0007093), regulation of mitotic cell cycle phase transition (GO:1901990), mitotic cell cycle process (GO:1903047), regulation of cell cycle phase transition (GO:1901987), and negative regulation of the mitotic cell cycle (GO:0045930). In contrast, the primary signaling pathways pertinent to Treg1 cells include the mitotic spindle assembly checkpoint signaling (GO:0007094), regulation of chromosome segregation (GO:0051983), negative regulation of chromosome organization (GO:2001251), regulation of mitotic sister chromatid separation (GO:0010965), and negative regulation of mitotic cell cycle phase transition (GO:1901991). Meanwhile, the predominant signaling pathways in Treg2 cells involve cytoplasmic translation (GO:0002181), macromolecule biosynthetic process (GO:0009059), cellular nitrogen compound biosynthetic process (GO:0044271), negative regulation of fatty acid transport (GO:2000192), and gene expression (GO:0010467). The principal signaling pathways implicated in DC1 cells encompass the negative regulation of the intrinsic apoptotic signaling pathway (GO:2001243), the regulation of DNA-templated transcription in response to stress (GO:0043620), the regulation of the intrinsic apoptotic signaling pathway (GO:2001242), the response to peptide hormone (GO:0043434), and the response to fluid shear stress (GO:0034405). Conversely, the predominant signaling pathways associated with DC2 cells include the cellular response to gamma radiation (GO:0071480), the cellular response to ionizing radiation (GO:0071479), the regulation of cell-matrix adhesion (GO:0001952), locomotor rhythm (GO:0045475), and the regulation of reactive oxygen species metabolic process (GO:2000377).

## Discussion

In this study, we employed single-cell RNA sequencing (scRNA-seq) to elucidate thymocyte maturation in *Tupaia belangeri yaoshanensis* across various age groups, marking the first such analysis in this species. Our research successfully identified the cellular composition of the thymus, thereby addressing a significant gap in single-cell thymic data for tree shrews, which are recognized as the closest model organisms to primates. This investigation not only provides essential data to support the utilization of tree shrews as experimental models in immunological studies but also offers novel insights into the development of the immune system in a model closely related to primates. We identified 20 distinct immune cell types within the thymus of tree shrews, which closely resemble those found in the thymus of pigs (22), yet are fewer than those identified in human thymic tissue (30). Furthermore, apart from dendritic cells, stromal cells were scarcely detected in tree shrews, contrasting with the reported abundance of stromal cells in the human thymus. Previous scRNA-seq analyses of the human thymus have demonstrated cellular heterogeneity within the thymic stroma, identifying potential key regulatory cell types that may play a pivotal role in thymic epithelial differentiation and thymocyte migration (50). The observed differences may be attributed to various factors, including the sample collection methods, sequencing depth, and the number of samples analyzed. Research has demonstrated that the diversity of cell types obtained through mechanical isolation of tissue cells is less than that achieved via enzymatic isolation. Nonetheless, the expression of surface markers on stem cells isolated through mechanical separation is more than double that observed in cells collected by enzymatic digestion (51). In this study, we employed the mechanical isolation technique to isolate thymocytes, as it offers significant advantages for single-cell sequencing analysis of tree shrews thymocytes, particularly when the marker molecules are not well-defined.

The thymus is a critical organ for maintaining homeostasis within the peripheral immune system. Within this mediastinal tissue, T cells undergo development and extensive education before being exported to the periphery to establish a functional and effective immune system. Aging induces significant alterations in the immune system, such as changes in the proportion and absolute number of T cells, which can diminish the immune response in older individuals. As the thymus undergoes atrophy, the epithelial spaces gradually disappear, and the perivascular space increasingly occupies the aging thymus. This results in a reduction in naïve T cells, an increase in peripheral late-differentiated memory T cells, and decreased migration of naïve T cells to the periphery (13, 52, 53). Our findings corroborate previous research by demonstrating an elevated presence of immature immune cells, including double-negative (DN) and double-positive (DP) cells, in the thymus of juvenile tree shrews. Conversely, as tree shrews age, there is a notable increase in the proportion of late-differentiated functional cells, such as regulatory T cells (Treg cells), in older individuals. These cells are essential for maintaining immune tolerance and preventing autoimmune responses.

In the thymus of tree shrews, we have identified a distinct population of B cells that play significant roles within this organ. B cells were distinguished by the specific expression of CD19, MS4A1, PAX5, CD40, and FCER2 (**Figure 1A**; **Supplementary Table S1-2**). This finding is consistent with previous reports indicating that pigs possess a rare population of B cells that localize in the thymic medulla upon maturation (54). A similar subset of B cells in mice has been shown to contribute to negative selection (55). Notably, the prevalence of B cells increases with age, reaching a peak in adulthood before declining in the elderly. This trend aligns with the exposure to antigens and the capacity to mount responses against them. The presence and function of B cells in the thymus have garnered considerable interest in immunological research. Traditionally, the thymus is recognized for its critical role in T cell development; however, recent studies have underscored the importance of other cell types, including B cells, in sustaining immune homeostasis and facilitating immune responses. Thymic B cells may contribute to the regulation of immune responses and the maintenance of tolerance. They have been implicated in the presentation of self-antigens, a process crucial for the negative selection of autoreactive T cells, thereby preventing autoimmune diseases. This function is supported by the expression of major histocompatibility complex (MHC) molecules on B cells, which are essential for antigen presentation and the induction of T cell tolerance (56).

In the investigation of regulatory T cells (Tregs) within the thymus of tree shrews, we identified two distinct cell populations characterized by typical Treg marker molecules. It is worth noting that the expression of the FOXP3 gene was not detected when we analyzed it using the first version of the reference genome. To eliminate misjudgment in the analysis process of this study and to find out why Treg cells do not express some key marker genes such as FOXP3, we re-grouped the cells using another version of the tree shrew reference genome (https://www.ncbi.nlm.nih.gov/datasets/genome/GCF_000334495.1/). In the analysis of the new tree shrew reference genome, we finally identified the key marker gene of FOXP3 in the corresponding Tregs cell population. Meanwhile, we found that the FOXP3 gene annotation was missing in the original version of the tree shrew reference genome. This is the reason why we did not find FOXP3 in our analysis. In fact, it does exist. We had reanalyzed the cell markers and supplemented them in **Supplementary Table S10**. The Tregs cells correspond to the 18th group of the new classification. Furthermore, the expression of the Foxp1 gene was detected in tree shrew Treg cells. Foxp1, a forkhead transcription factor and a homolog of FOXP3, plays a critical role in maintaining optimal expression of FOXP3 specifically in extrathymic Treg (iTreg) cells. The deletion of Foxp1 leads to a gradual loss of FOXP3 in iTreg cells, resulting in a significantly diminished Nrp1−Helios− iTreg population and increased intestinal inflammation in aged mice (57). This observation diverges from findings in humans and other species. For instance, in the human thymus, Treg cell development is also associated with FOXP3 expression, although the precise developmental stage and underlying mechanisms remain subjects of ongoing research (58). Some studies have reported that Treg cells commence differentiation during the double-positive phase, indicating that, in certain contexts, Treg cell development may precede FOXP3 expression (59). Furthermore, our study suggests that B cells within the thymus may contribute to the development and proliferation of Treg cells, potentially through mechanisms involving cell-to-cell contact via MHC II and co-stimulatory molecule pathways (60). These findings imply that Treg cell development may be modulated by multiple factors, including intercellular interactions and specific signaling pathways. In conclusion, compared with canonical human mature regulatory T cells (Tregs), tree shrew Treg cells highly express the FOXP3 gene. It is necessary to explain that some key genes are indispensable in the differentiation and development of Treg cells, which enhances our understanding of the developmental pathways of Treg cells among different species and clarifies their important role in immune regulation.

In the thymus of tree shrews, the presence and function of KLF2+ CXCR3+ CD4 T cells have garnered significant scholarly interest. The detection of KLF2+ CXCR3+ CD4 T cells in the thymus, particularly during infancy, implies a potentially pivotal role for these cells in the initial development of the immune system. Empirical studies have demonstrated that memory CD4+ T cells can originate in the fetal gut, suggesting that the formation of memory T cells may occur during early developmental stages, even in the absence of exposure to exogenous antigens (61). These findings underscore the multifaceted role of the thymus in immune system development, particularly concerning the generation and maintenance of memory T cells. Elucidating these mechanisms may enhance our understanding of the establishment of the immune system in early life and offer novel insights into the treatment of immune-related diseases.

Pseudotime analysis is a widely used method for single-cell RNA sequencing (scRNA-seq) data to infer the dynamic trajectories of cells during development or differentiation. This study found that the sequencing order of tree shrew thymocytes was basically highly consistent with the known marker genes and stage-specific transcription factors. However, in this analysis, we found that the development time sequence of some cells in the pseudo-temporal analysis results still deviated from the theory to some extent. One of the main reasons we believe is that the analysis process included cells at different stages of the cell cycle. Specifically, the positions of cell populations in the early stages of the cell cycle (such as cells in the G1 phase or the early S phase) in the pseudo-time trajectory have deviated, resulting in the same type of cells being wrongly mapped to different time points or branches.

The conservation of thymocyte signatures across diverse species, including humans, pigs, and tree shrews, underscores the evolutionary stability of immune system components. Elucidating the conservation of these signatures offers valuable insights into the fundamental mechanisms underpinning immune function and development across species. Empirical research has demonstrated a significant degree of conservation in immune responses among nonhuman primates and other mammals. For example, a study examining cell-intrinsic immune responses across a broad spectrum of nonhuman primate species, including humans, identified a high degree of conservation in the functional outcomes of immune responses. This finding suggests that core immune mechanisms are preserved across these species (62). Such conservation is pivotal for comprehending host-pathogen interactions and the evolutionary pressures influencing immune system development. Specifically, in the context of thymocyte development, the conservation of particular gene expression profiles and regulatory elements is vital for sustaining the integrity of T cell development.

Tree shrews, which share a close evolutionary relationship with primates, have been demonstrated to possess immune system components that exhibit remarkable similarity to those of humans, thereby establishing them as a valuable model for the investigation of human diseases (63). In our comparative analysis of gene expression across species, we observed that the gene expression profiles of thymic immune cells in tree shrews are largely conserved relative to those in humans and pigs (**Figure 4**). This conservation is particularly pronounced in key marker genes that play essential roles in immune function across diverse species. For instance, research has indicated that the chemokine CXCL8 and its receptors CXCR1/2 in tree shrews are highly conserved both structurally and functionally when compared to humans, implying that tree shrews may possess chemotactic capabilities akin to those of primates (64). Nevertheless, despite the high degree of conservation, certain genes exhibit differential expression across species. For example, when comparing gene expression in primary cells from humans, pigs, and tree shrews, some genes display significant differences in expression levels and temporal patterns among the different species. This disparity may be attributed to evolutionary modifications in intercellular communication mechanisms across species (65). These findings suggest that, despite the conservation of certain key genes, distinct species continue to exhibit unique evolutionary trajectories in gene expression.

To elucidate the transcriptional alterations associated with aging in the thymus of tree shrews, we conducted an analysis of the coefficient of variation (CV) within the gene population. Our findings reveal that the gene-based coefficients of variation initially increase and subsequently decrease with age. Notably, the coefficient of variation in adult tree shrews was highest during infancy, with a statistically significant difference observed. This phenomenon may be attributed to the heightened intercellular transcriptional variability that accompanies aging. Previous research has demonstrated that aging disrupts the initiation of core gene activation programs and enhances expression heterogeneity among cell populations (66). Furthermore, aging is linked to alterations in gene expression across various tissues, which may manifest in distinct patterns and intensities depending on the tissue type (67). For instance, studies have indicated that selection pressure on genes diminishes with age, potentially leading to changes in gene expression and evolutionary patterns at different life stages (68). These findings underscore the complexity and diversity of gene expression changes during aging, suggesting the involvement of multiple biological processes and interactions among tissues.

In the investigation of gene expression in tree shrews, it has been observed that gene expression patterns during infancy and senescence exhibit notable differences in their roles in regulating aging. Tree shrews in their infancy generally demonstrate elevated expression levels of genes involved in aging regulation, with these expressions progressively diminishing as the organisms age. This pattern may be attributed to the rapid growth and developmental demands characteristic of infancy, during which cell proliferation and differentiation activities are heightened, necessitating increased gene regulation to sustain physiological equilibrium. Parallel studies on Drosophila melanogaster have revealed that numerous genes associated with muscle development and maintenance are markedly downregulated in older flies. These genes are active in juvenile flies, particularly within the direct and indirect flight muscles where they are uniquely expressed (69). This evidence suggests that the age-related decline in the expression of these genes may be linked to a deterioration in muscle function. Conversely, the gene expression profiles of aged tree shrews exhibit a distinct pattern: genes involved in the regulation of aging demonstrate increased expression as the organisms advance in age. This alteration is potentially linked to physiological modifications associated with aging, such as enhanced cellular repair and antioxidant defense mechanisms, which serve to mitigate damage and stress induced by the aging process (70). Furthermore, research suggests that alterations in gene expression during old age may be connected to the regulation of the immune system, particularly genes that govern immune function and inflammatory responses. The upregulation of these genes may contribute to resistance against age-related diseases (71). In conclusion, the variations in gene expression observed in tree shrews across different life stages reflect their adaptive responses to environmental and physiological challenges encountered during growth, development, and aging. These findings not only enhance our comprehension of tree shrews as model organisms in aging research but also offer a foundation for further investigation into aging mechanisms in other species.

Despite the extensive transcriptional analysis of thymus development presented in this study, contemporary research predominantly emphasizes lymphocytes, with comparatively less focus on the supporting cell populations. The thymus, a complex primary lymphoid organ, serves as an essential site for lymphocyte development and encompasses a variety of supporting cells, including thymic epithelial cells, endothelial cells, interstitial cells, and dendritic cells, all of which are integral to the establishment and maintenance of the thymic microenvironment (72). To achieve a more thorough understanding of thymic function and development, it is imperative that future research endeavors enhance the collection and analysis of thymus samples and cell types from tree shrews. Utilizing single-cell RNA sequencing technology will enable the elucidation of the heterogeneity among thymic stromal cells and facilitate the identification of potential key regulatory factors, which are vital for comprehending thymic epithelial cell differentiation and thymocyte migration.

## Data Availability

The datasets presented in this study can be found in online repositories. The names of the repository/repositories and accession number can be found below: https://ngdc.cncb.ac.cn/gsa. (GSA: CRA022608).
