# Supplementary material for: Decoding thymic development: a single-cell atlas of tree shrew immunity
Source: Front Immunol. 2026 Jan 16;16:1689906. doi: 10.3389/fimmu.2025.1689906 (PMC12855143; doi:10.3389/fimmu.2025.1689906)
Supplement: Supplementary file 2 [file DataSheet1.pdf]

Supplemental information

Decoding Thymic Development: A Single-Cell Atlas of Tree Shrew Immunity

Haibo Tang, Yunlin He, Lifeng Zhang, Yingying Cao, Baoying Li, Liang Liang, Chengxia Yun, Junyu Tao, Shanshan Zhai, Zhuxin Li, Yinghan Dai,Yanling Hu, Jing Leng

Supplemental materials

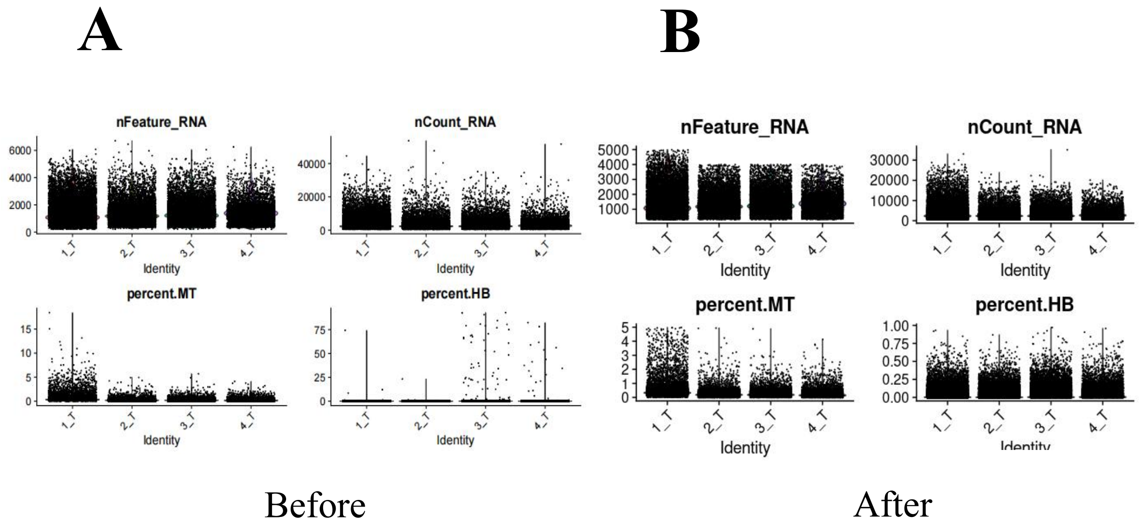

Figure S1. After quality control of scRNA-seq, 36,183 cells were identifed.
A positive correlation was observed between nCount_RNA, indicating the number of unique molecular identifiers (UMIs), and nFeature_RNA, representing the number of genes. The expression profiles of each sample prior to quality control are illustrated in Figure S1A, while those following quality control are depicted in Figure S1B.

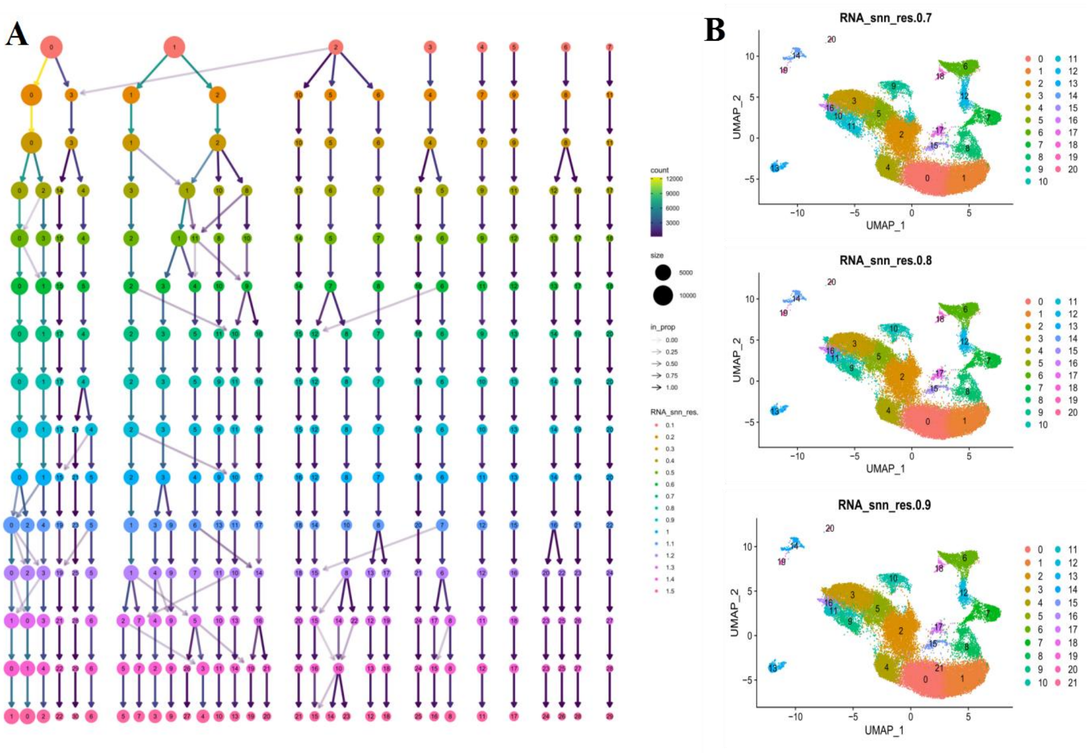

Figure S2. Visualize UMAP images with a resolution range of 0.1 to 1.5.

Unsupervised clustering of cells for quality control to identify various clusters. (A) various clusters were visualized using a UMAP plot with a resolution range of 0.1 to 1.5. (B) The Uniform Manifold Approximation and Projection (UMAP) visualization of porcine thymus cell types is presented, with cell clusters distinguished by color. These clusters were identified utilizing the graph-based Louvain algorithm at resolutions of 0.7, 0.8, and 0.9.

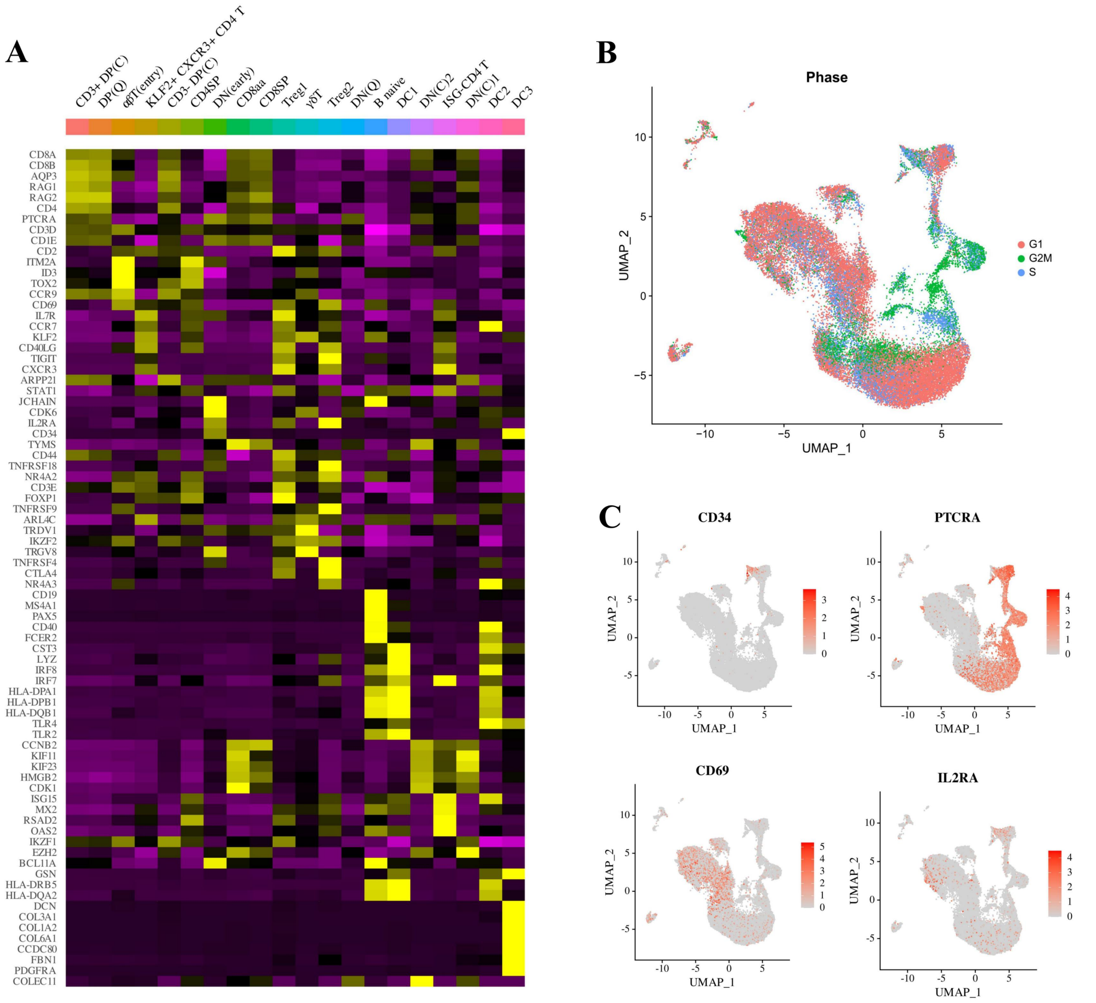

Figure S3. presents the transcriptional profile of thymocytes in tree shrews, as referenced in Figure 1. (A) The heatmap illustrates the row-scaled mean expression levels of five marker genes across each cluster (as delineated in Figure 1A). (B) UMAP plot showing cell cycling phases in tree shrews thymocytes. The same UMAP plot is used to show cells with high expression of CD34, PTCRA, CD69, and IL2RA.

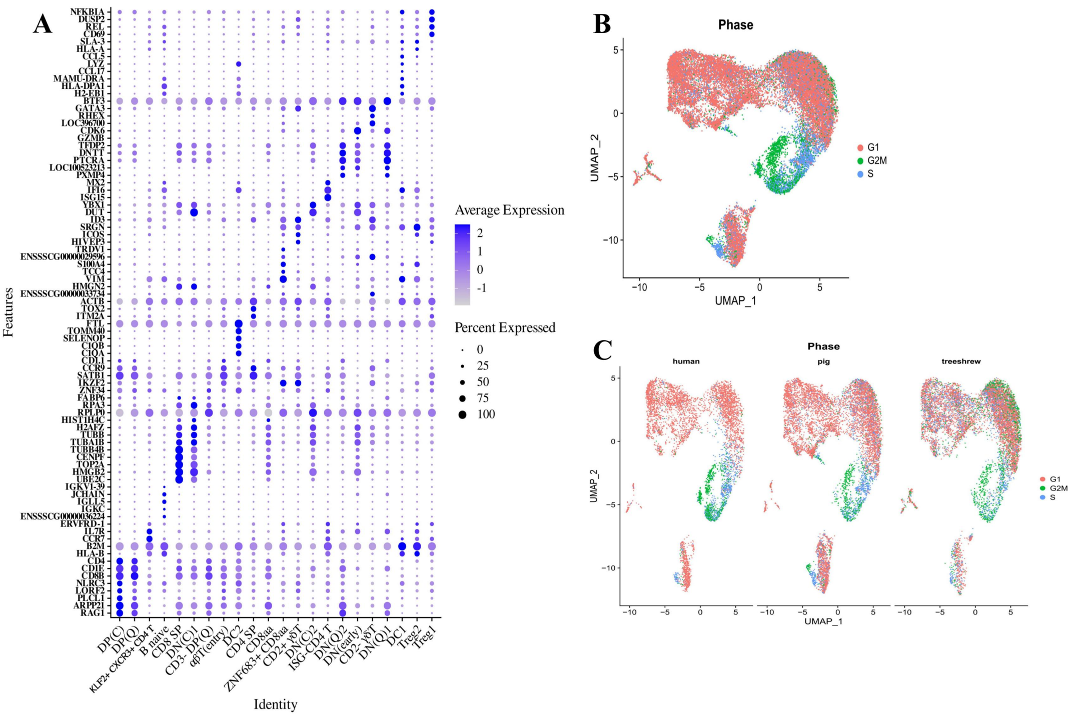

Figure S4. Transcriptional Profiling of Tree Shrew Thymocytes in Relation to Figure 4A. (A) The heatmap presents the row-scaled mean expression levels of five marker genes across each cluster, as identified in Figure 4A. (B) UMAP plot depicting the cell cycle phases in thymocytes across different species. (C) UMAP plot illustrating the cell cycle phases in thymocytes of humans, pigs, and tree shrews, respectively.

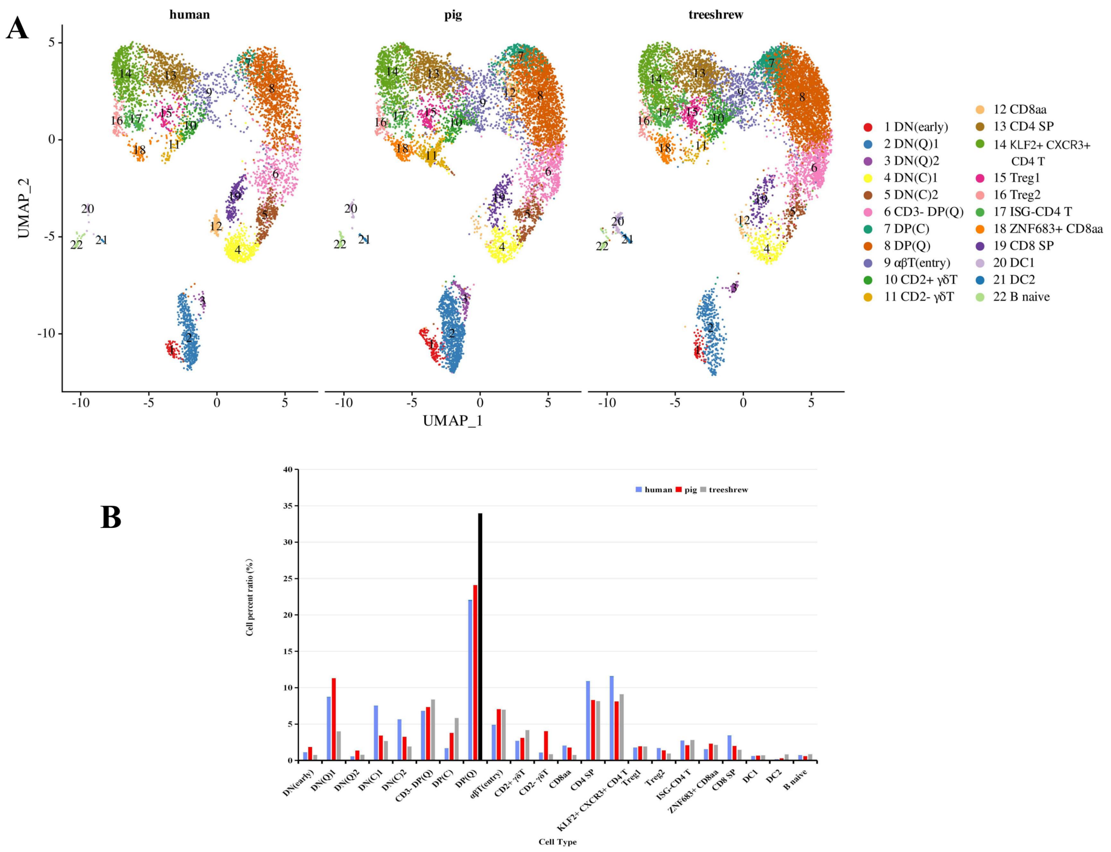

Figure S5. Comparative Thymocyte Distribution Among Species.

(A) The Uniform Manifold Approximation and Projection (UMAP) visualization demonstrates the distribution of thymocyte types across multiple species, with distinct clusters identified through color coding. Cluster identification was achieved using the Louvain algorithm with a resolution parameter set at 1.3. (B) A graphical representation illustrates the proportion of thymocytes relative to the total cell count in the thymus across different species.

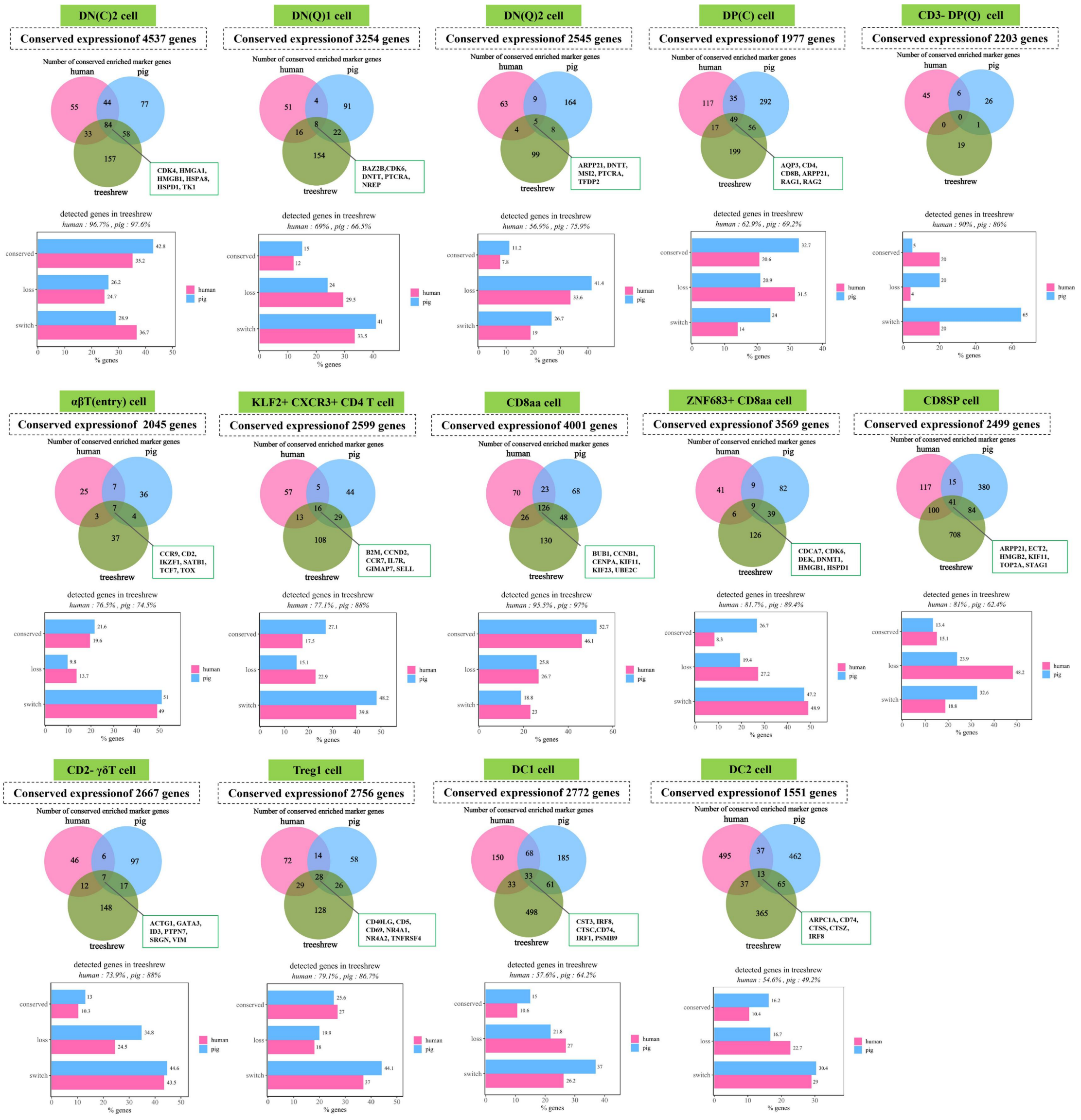

Figure S6. Conservation of Marker Signatures in Thymocytes of Humans, Pigs, and Tree Shrews. The upper panel presents a Venn diagram illustrating the overlap of enriched marker genes across species for each cell type. Only marker genes that are mappable across species are included. Selected known overlapping cell type markers and the number of genes exhibiting conserved expression are highlighted. Enriched marker genes are characterized as those expressed in more than 5% of cells within the corresponding cell type and demonstrating increased expression relative to all other cell types (log2-fold change > 0.5). The lower panel depicts the conservation of tree shrew-enriched marker genes in human and pig cell types. The percentage of tree shrew-enriched marker genes that are expressed or detected is indicated. Categories

include: "Conserved," where the enriched marker is present in the same cell type as in tree shrews; "Loss," where the gene is detected but not as an enriched marker; and "Switch," where the enriched marker is present in a different cell type compared to tree shrews.

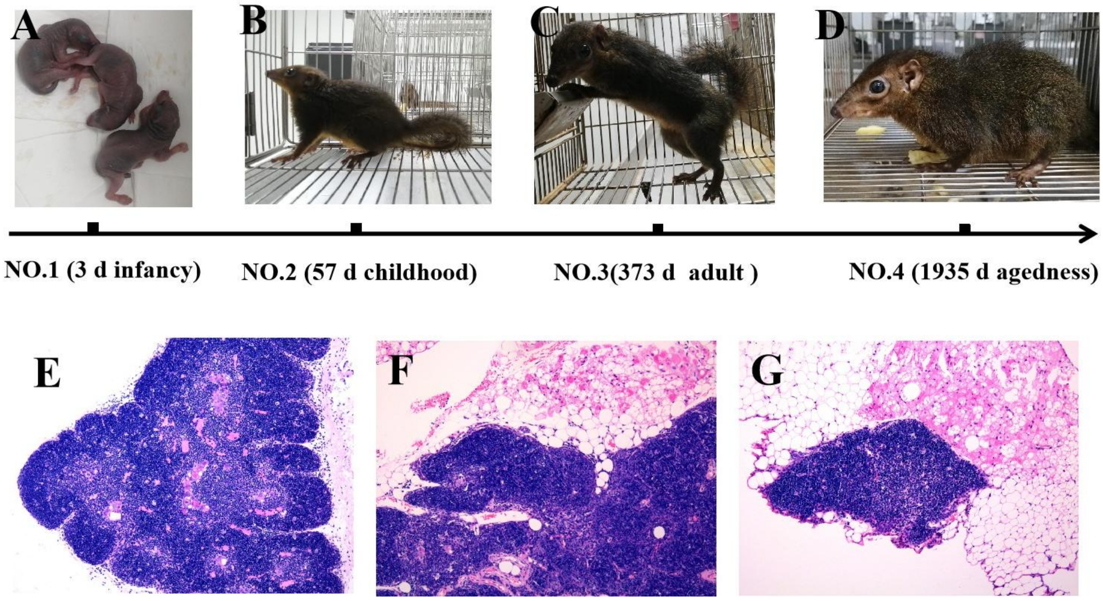

Figure S7. Photographic and Histological Analysis of Tree Shrews.

(A) Photographic documentation of tree shrews at various developmental stages 3 days, 57 days, 1 year, and 5 years and 3 months were conducted in this study. (B) Histological examination through Hematoxylin and Eosin (H&E) staining of thymus tissue from tree shrews at 3 days, 1 year, and 5 years and 3 months of age.

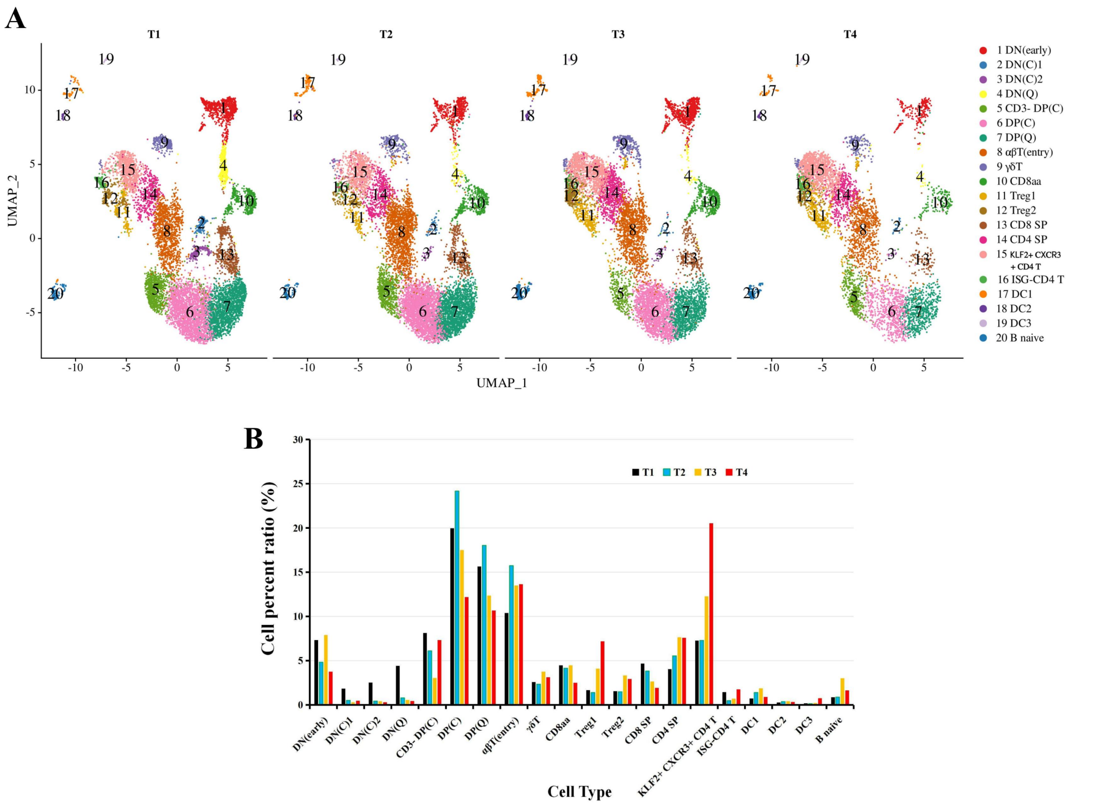

Figure S8. The Distribution of Thymus Cells in Tree Shrews.

(A) Unified Manifold Approximation and Projection (UMAP) visualization depicting thymocyte types in tree shrews across various ages, employing cluster color coding. The Leuven algorithm, with a resolution parameter set at 0.8, was utilized for cluster identification. (B) A graphical representation illustrating the proportion of thymocytes relative to the total cell population in tree shrews of differing ages.

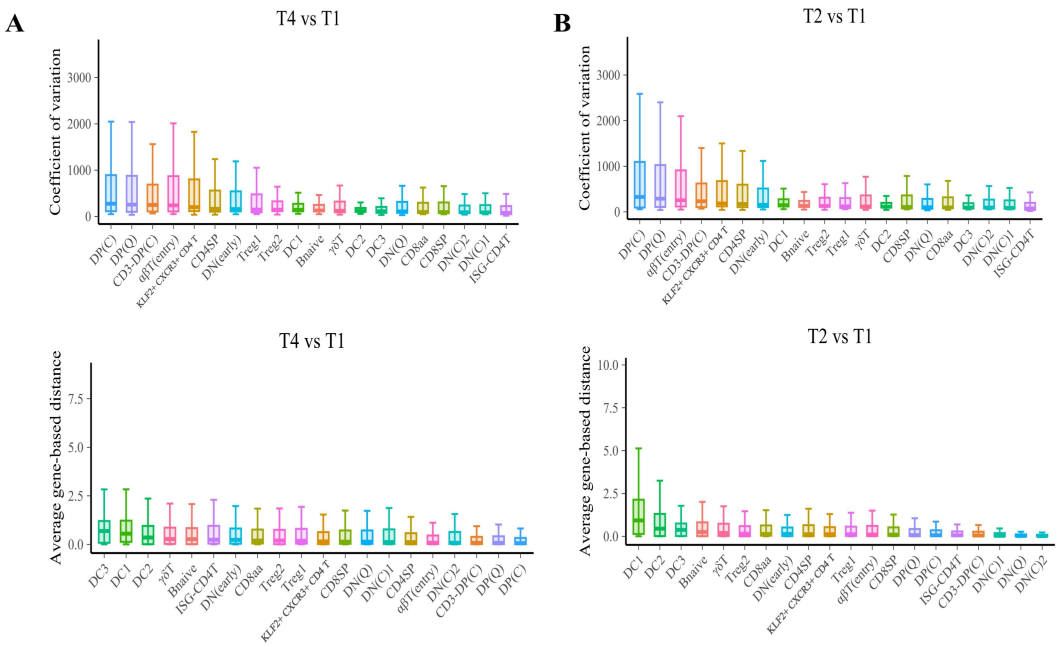

Figure S9. presents an analysis of the coefficient of variation (CV) of thymocytes in tree shrews across different cell types.

Panels (A) and (B) feature box plots that compare the CV of each cell type at 5 years and 3 months with that at 3 days of age, and at 57 days with that at 3 days, respectively. These plots display the median and interquartile range (25%-75%), with whiskers extending to 1.5 times the interquartile range. Panels (C) and (D) illustrate the calculation of the coefficient of variation as the standard deviation of the distances between genes within cell clusters, normalized by the mean of these distances, for the ages corresponding to those in panels (A) and (B).
